# Compositional diversity of rehabilitated tropical lands supports multiple ecosystem services and buffers uncertainties

**DOI:** 10.1038/ncomms11877

**Published:** 2016-06-13

**Authors:** Thomas Knoke, Carola Paul, Patrick Hildebrandt, Baltazar Calvas, Luz Maria Castro, Fabian Härtl, Martin Döllerer, Ute Hamer, David Windhorst, Yolanda F. Wiersma, Giulia F. Curatola Fernández, Wolfgang A. Obermeier, Julia Adams, Lutz Breuer, Reinhard Mosandl, Erwin Beck, Michael Weber, Bernd Stimm, Wolfgang Haber, Christine Fürst, Jörg Bendix

**Affiliations:** 1 Institute of Forest Management, Department of Ecology and Ecosystem Management, TUM School of Life Sciences Weihenstephan, Technische Universität München, 85354 Freising, Germany; 2 Institute of Silviculture, Department of Ecology and Ecosystem Management, TUM School of Life Sciences Weihenstephan, Technische Universität München, 85354 Freising, Germany; 3 Departamento de Economía, Universidad Técnica Particular de Loja, 1101608 Loja, Ecuador; 4 Institute of Landscape Ecology, University of Muenster, 48149 Münster, Germany; 5 Institute for Landscape Ecology and Resources Management, Justus Liebig University Giessen, 35392 Giessen, Germany; 6 Department of Biology, Memorial University, St John's, NL, Canada A1B 3X9; 7 Laboratory for Climatology and Remote Sensing (LCRS), Faculty of Geography, University of Marburg, 35032 Marburg, Germany; 8 Department of Plant Physiology and Bayreuth Centre of Ecology and Environmental Research, University of Bayreuth, 95440 Bayreuth, Germany; 9 Chair of Terrestrial Ecology, Department of Ecology and Ecosystem Management, TUM School of Life Sciences Weihenstephan, Technische Universität München, 85354 Freising, Germany; 10 Karlsruhe Institute of Technology (KIT), Institute of Meteorology and Climate Research-Atmospheric Environmental Research (IMK-IFU), Campus Alpin, 82467 Garmisch-Partenkirchen, Germany

## Abstract

High landscape diversity is assumed to increase the number and level of ecosystem services. However, the interactions between ecosystem service provision, disturbance and landscape composition are poorly understood. Here we present a novel approach to include uncertainty in the optimization of land allocation for improving the provision of multiple ecosystem services. We refer to the rehabilitation of abandoned agricultural lands in Ecuador including two types of both afforestation and pasture rehabilitation, together with a succession option. Our results show that high compositional landscape diversity supports multiple ecosystem services (multifunction effect). This implicitly provides a buffer against uncertainty. Our work shows that active integration of uncertainty is only important when optimizing single or highly correlated ecosystem services and that the multifunction effect on landscape diversity is stronger than the uncertainty effect. This is an important insight to support a land-use planning based on ecosystem services.

## Abstract (web summary)

Land use becomes more diverse when it considers uncertain interactions of multiple ecosystem services. Here, Knoke and colleagues show that uncertainty plays a larger role if ecosystem services are optimized only for a single service, or if services correlate.

## Graphical abstract

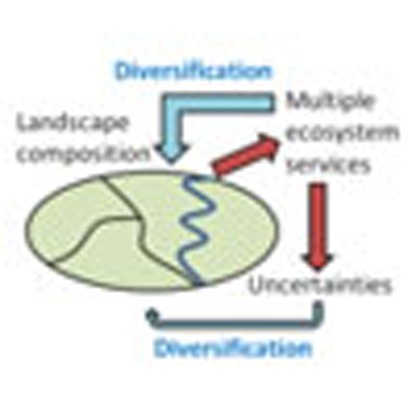

The compositional diversity of a landscape has crucial consequences for the level and stability of ecosystem services. Compositional diversity can be quantified by different landscape metrics (including familiar indices such as Shannon's diversity^1^) that account for the number and proportions of land-use/cover types^2^. Ecosystem processes and functions are less adversely affected by disturbance when landscapes have high compositional diversity^1^,^3^. Diversifying crop composition on farms^4^,^5^ and tree species in forests^6^ can also increase levels of multiple ecosystem services. Crop diversification furthermore protects farmers against economic uncertainties and market shocks^7^,^8^,^9^,^10^. Consequently, it is important to consider how to incorporate landscape diversification in initiatives to rehabilitate the abandoned agricultural lands.

Abandoned agricultural lands are abundant worldwide with an estimated extent between 385 and 472 million hectare (ref. 11). Rehabilitation of these lands means re-establishing productive functions of the ecosystem and often includes reintroducing some of the original flora and fauna^12^. Rehabilitation can mitigate the loss of tropical forests, because it allows agricultural or forestry production to resume on abandoned lands, reducing the need to expand into natural forest^13^,^14^. However, local efforts to rehabilitate or restore abandoned lands often focus only on single, promising land-use/cover types, particularly afforestation, and disregard options for diversification. This may lead to unfavourable outcomes. For example, Derak and Cortina^15^ have shown that concentrating on a single option only does not necessarily improve the whole suite of ecosystem services. Rehabilitation initiatives that aim to create landscapes with high compositional diversity could be a better alternative.

Despite empirical evidence supporting the advantages of high landscape diversity, the mechanisms behind land allocation, the resulting multiple ecosystem services and various dimensions of uncertainty are not yet well understood. For example, we do not know the exact levels of current or future ecosystem services, and should therefore consider possible variations in the expected levels of these services. For systematic land-use planning, an improved understanding of how the uncertainties of ecosystem services, on one hand, and the demand for multiple ecosystem services, on the other hand, influence the ‘optimal' allocation of land is necessary. For example, it has been shown that optimized land allocation can mitigate trade-offs between multiple services^16^.

Here we adopt a new approach based on uncertainty spaces for dealing with uncertainties/disturbances when optimizing the allocation of land to alternative land-use/cover options^17^. We combine this approach with multiobjective optimization^18^,^19^,^20^,^21^,^22^,^23^. This not only helps to address multiple desired ecosystem services, but also accounts for the often unclear or unknown preferences of current and future generations^24^. Our study defines the uncertainty of ecosystem services as possible negative or positive deviations from the expected levels (that is, disturbance from these levels), when such services are quantified by measurable associated variables (indicators). Deviations may occur for various reasons, such as measuring, sampling or model prediction errors, or because of unanticipated future environmental change, unpredictable market development or unexpected damage by calamities^25^.

Our optimization is developed to support the rehabilitation of abandoned agricultural lands in Ecuador. It considers the option of leaving pastures abandoned (as natural succession areas) plus four active options for rehabilitation: afforestation either with the native *Alnus acuminata* or the exotic *Pinus patula*, and repasturization (re-establishing pastures) either with subsequent low-input or intense management. We structure our study around two aspects: (1) the impact of multiple ecosystem services on optimal land allocation (multifunction effect); and (2) the impact of increasing levels of possible deviation from recorded/modelled indicators on the optimal allocation of land (uncertainty effect). With respect to the multifunction effect, we expect that the provision of multiple ecosystem services (represented by ecosystem service indicators) can be improved only by considering many land-use/cover types and not a single one. Thus, the compositional landscape diversity should increase with the number of indicators considered. Integrating possible deviations from the initial indicators (uncertainty effect) into the optimization will support high landscape diversity as well, because deviations will be different for several land-use/cover types. Consequently, we expect both aspects to drive landscape composition in the same direction (Fig. 1), meaning that landscape diversity will increase as more ecosystem services are considered and with growing uncertainty.

Throughout this paper we use the term ‘ecosystem services' in a broader sense, defined as all ecosystem aspects used actively or passively to support human well-being^26^. Depending on the purpose, these ecosystem aspects can be classified as ecosystem processes, functions, services, benefits or values^27^,^28^. Our paper distinguishes between two types of ecosystem service indicators, namely ‘ecological' and ‘socioeconomic' indicators^14^. The ecological indicators are associated with carbon relationships, climatic or hydrological regulation, and soil properties. They describe ecosystem processes or functions, and influence the suitability of the site for, and sustainability of, different land-use/cover types. For instance, biomass production is a prerequisite for subsequent provision of food, fodder or timber as a service. In contrast, socioeconomic indicators address the more direct benefits for farmers, such as economic return (quantified as net present value of net revenues), payback periods (time until invested money is received back) or the general acceptance of the rehabilitation options by farmers. To describe the results we will mainly refer to land-cover rather than land-use types, because unmanaged areas left abandoned may play an important role in new concepts of land allocation. Finally, we will focus on the optimization of the diversity of land-cover types (compositional complexity), and exclude the impact of landscape configuration.

Our results show that high landscape compositional diversity is realized when optimization aims to improve levels of multiple ecosystem services, even when ignoring uncertainties. Consequently, the aim of providing multiple ecosystem services is the major driver of landscape diversity, rather than the uncertainties involved. However, this is only true when the indicators used are uncorrelated. Considering highly correlated indicators leads to homogeneous landscapes, dominated by afforestation. Such landscapes do not buffer against disturbance. Thus, when indicators are correlated, integrating uncertainties into the optimization is essential to avoid unfavourable results under perturbations. Such consideration of uncertainties leads to converging landscape composition, irrespective of the degree of correlation between indicators.

## Results

### Multifunction effect

An extensive set of 22 indicators covering all categories of ecosystem services as defined by the Millennium Ecosystem Assessment^29^ forms the basis for our optimization. Indicators are associated with supporting (biomass production and soil quality) and regulating functions (carbon, climate and hydrology), as well as with provisioning services (timber and food) and social benefits (acceptance by the local people) (Table 1, for details see Knoke *et al*.^14^ and Supplementary Methods).

Our optimization considers a range of indicator levels under uncertainty through possible deviations from the initial indicator values, as depicted in Fig. 2. The optimization approach (Methods) allocates land to rehabilitation options with the aim to minimize low performance of landscape level indicators (Fig. 3). This optimization increases the levels of multiple ecosystem service indicators, particularly those with the lowest original levels (Fig. 3), and leads to a quite complex optimal landscape composition. The resulting structure of the anthropogenic landscape mosaic (hereafter called a ‘landscape portfolio') is 24% abandoned areas, 21% *Alnus*, 25% *Pinus*, 10% low-input pastures and 20% intense pastures, given a level of uncertainty of *f*_U_=2 (meaning that the considered deviation from the recorded/modelled indicator is twice as large as its s.e.m., see Methods). Shannon's diversity of land-cover types is 1.57 for this landscape portfolio, which is close to the theoretical maximum diversity of 1.61. This result underlines a strong multifunction effect on landscape complexity.

Random experiments drawing various combinations of 4, 8, 12 and 16 indicators out of 22 (with 50% ecological and 50% socioeconomic indicators in each experiment) show the impact of the number of indicators considered (Fig. 4). The obtained composition of land-cover types is most variable and thus volatile for a small number of four randomly selected indicators, but it stabilizes when integrating 8–12 indicators into the optimization. For example, with various combinations of only eight indicators all five land-cover types are represented in almost all repetitions.

**Figure 1:**
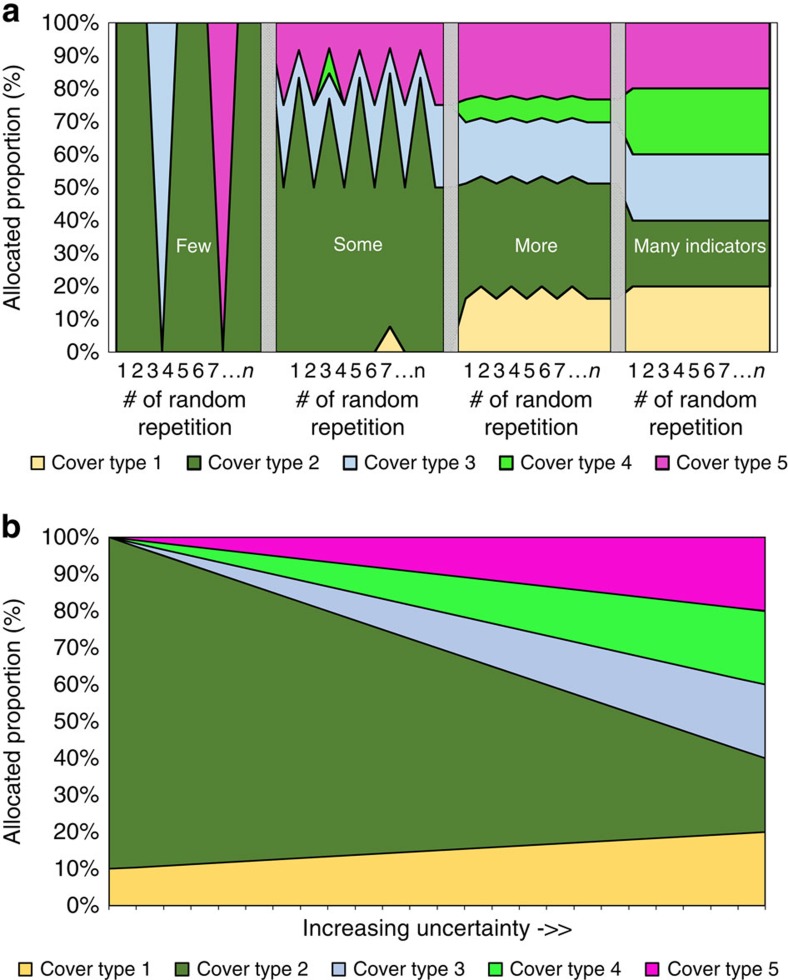
Schematic overview of theoretically expected effects. (**a**) Possible impact of multiple ecosystem service indicators, and (**b**) possible impact of indicator uncertainty on the allocation of land proportions. In **a**, landscape composition depends on the actual indicators selected, which is often subject to some randomness but also to pragmatic aspects, such as availability. Using only a few indicators may lead to relatively homogenous landscapes, because improved levels of a small number of indicators can be achieved by single land-cover types. Depending on the indicators chosen, landscape composition may change completely (for example, repetition #3 and #7 for few indicators). However, as more ecosystem service indicators are considered, more diversified landscape compositions may occur to address the demand for multiple ecosystem services. In **b**, only one or a few land-cover types may dominate when uncertainty is excluded from the optimization, which may be those with good performance for several indicators. With increasing uncertainty the landscape may tend towards greater diversification to buffer the uncertainties of single land-cover options.

Interestingly, when considering eight indicators or more the land allocation is hardly influenced by the number of indicators included. Only the optimization with four indicators results in less abandoned lands (11%) and greater proportions of *Alnus* (30%) and intense pasture (25%) compared with the optimization based on more indicators. However, even with only four indicators we obtain quite diversified, albeit highly variable, landscapes. In contrast to our expectation (Fig. 1a), each of the 10 random repetitions includes 4 or 5 land-cover types. This is in part an interaction effect due to the uncertainty considered, which also supports landscape diversification. When excluding uncertainty, the landscape portfolios still comprise always two or three land-cover types. This pattern and particularly the stability of the average landscape composition for optimizations with eight or more indicators, is a result of our allocation approach. It considers each landscape-level indicator separately, while requiring the highest possible minimum level for each indicator. Consequently, the landscape-level indicators are not averaged or summed; this means that averaging effects among the indicators do not affect the interplay between indicators and landscape composition. Our obtained Shannon diversities for the average landscape composition are more or less constant for eight indicators and more (varying from 1.54 to 1.55), indicating no change in landscape diversity with increasing numbers of indicators included in the optimization. In conclusion, merely increasing the number of indicators does not lead to systematically altered landscape portfolios, when considering eight or more indicators. However, increasing the number of indicators reduces the variability of the resulting landscape composition.

The optimization based on eight or more indicators comes with certain trade-offs. It leads to a moderate decrease in the achieved average performance for aggregated indicators, when compared with an optimization with four indicators (Table 2). Considering eight or more indicators helps to achieve a balanced provision of multiple uncertain ecosystem services with improved minimum levels for multiple ecosystem indicators. Compared with optimization based on four indicators, considering eight or more indicators increases the proportion of land allocated to abandoned areas from 11 to 24%. This reduces the proportion of *Alnus* from 30% to 21% and that of intense pasture from 25 to 21%. As a consequence, the average indicators for aggregated climate and hydrological regulation, net present value, and preferences decrease, while payback periods improve and carbon relations and soil quality remain constant.

### Uncertainty effect

**Figure 2:**
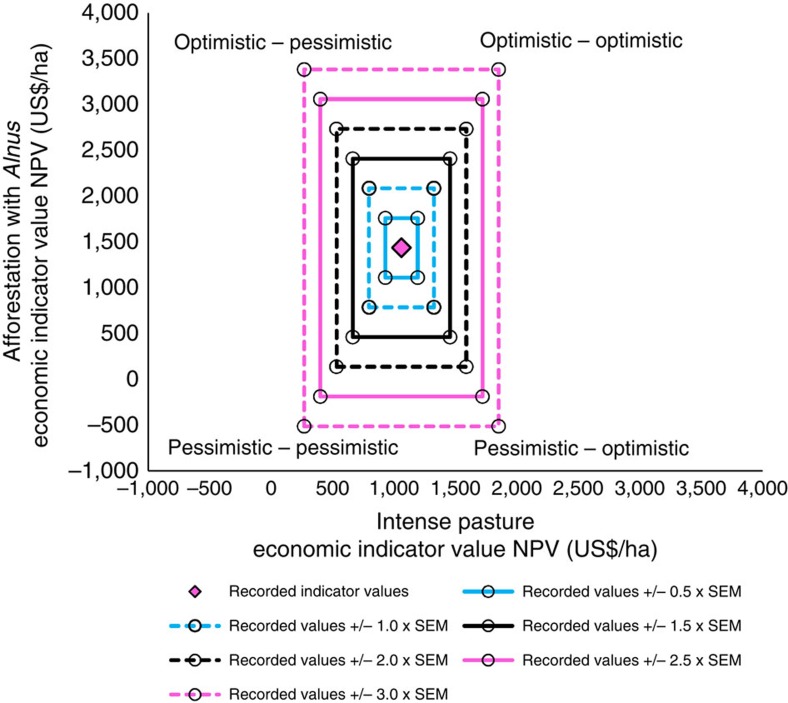
Example of a non-stochastic consideration of possible deviations. We refer to one of the economic indicators (net present value of net revenues, NPV, 5% discount rate) for two rehabilitation options, *Alnus* afforestation and intense pasture. Deviations for two rehabilitation options form an uncertainty area (see Knoke *et al*.^17^), while the actual investigation considers five options, which form five-dimensional uncertainty spaces. Starting from the recorded/modelled values (magenta diamond), box uncertainty spaces are constructed by means of adding/subtracting possible deviations (proportional to the measured s.e.m.) from the recorded indicator values. We enlarged the size of the boxes to consider different levels of uncertainty, *f*_U_, from 0.5 to 3.0 times the s.e.m. Minimizing the distance between the level of the indicator actually achieved and the maximum (100% of potential) level for that indicator (Fig. 3) considers all uncertainty combinations of indicator values included in the uncertainty boxes. Addressing the parameter combinations at the corner points of the rectangular boxes ensures all uncertainty combinations are considered. These combinations form the discrete uncertainty scenarios considered in equation (1) (see Methods).

We consider disturbance for each ecosystem service indicator by incorporating possible positive or negative deviations from recorded/modelled indicator values into the optimization (Fig. 2). In addition, we incorporate a large range of ecosystem indicators without any weighting or opportunity of compensation among them to acknowledge the prevailing uncertainty of human preferences^24^, which are often mutable, particularly over long timeframes^30^ (preferring specific services is analysed in ‘Sensitivity considerations').

In the investigations above, the optimized landscape portfolio only considers one level of uncertainty (*f*_U_=2), whereas here we vary the level of uncertainty. We have seen that a landscape that simultaneously increases multiple indicators consists of a quite diversified, relatively balanced landscape portfolio. Even when excluding uncertainty for each indicator, a landscape optimized to provide a balanced bundle of ecosystem services is already diversified (Fig. 5a, uncertainty level *f*_U_=0), with only low-input pastures excluded. This diversified landscape provides an implicit and effective buffer against uncertainty.

Increasing indicator uncertainty leads to only slight changes in land-cover patterns, if both socioeconomic and ecological indicators are considered. For example, at a very high uncertainty level of *f*_U_=3, the portfolio consists of 19% abandoned areas, 23% *Alnus*, 24% *Pinus*, 13% low-input pastures and 21% intense pastures (Fig. 5a). This landscape is slightly more diversified compared with the landscape portfolio obtained for lower levels of uncertainty. Thus, the simulated diversity of the rehabilitated abandoned lands increases with rising levels of uncertainty. However, this uncertainty effect is much less pronounced than expected (Fig. 1b).

The impact of the uncertainty effect on landscape composition thus appears to be less important compared with the multifunction effect. If the considered land-cover types all provide different services, then high compositional diversity appears to be an intuitive result when demanding multiple services. For example, the afforestation options will deliver favourable climatic regulation services, while intense pasture offers relatively low payback periods.

However, high landscape diversification to increase multiple ecosystem services is only a robust result when the ecosystem service indicators used for optimization are uncorrelated. An optimization example with only socioeconomic indicators shows the consequences of using correlated indicators (socioeconomic indicators show high correlation, Supplementary Table 1). When uncertainty is excluded in this example, rehabilitation only considers two options (Fig. 5b): *Alnus* afforestation and leaving area abandoned. This tendency towards homogenous landscapes is even more pronounced when we systematically combine the highly correlated indicators (Supplementary Fig. 1).

When using correlated indicators, it is mainly the uncertainty that drives the diversification of landscape portfolios. The optimization results obtained therefore resemble those of single-objective optimization in many cases (Supplementary Figs 2–9). However, general single-objective optimization may also lead to a single land-cover type, even if uncertainty is considered; this is the case for 6 of the 22 indicators. In conclusion, when indicators are highly correlated or single objectives are used, it is necessary to consider uncertainties, to avoid reduced landscape diversity, which will not buffer against uncertainty.

In contrast, the optimization with uncorrelated ecological indicators leads to relatively complex landscape portfolios very similar to those obtained from optimizations using all indicators (Fig. 5c). Moreover, the resulting landscape portfolios do not change greatly with the additional consideration of the socioeconomic indicators in our study.

As uncertainty increases, the optimized land proportions converge to a maximally diversified landscape portfolio, as shown by the Bray–Curtis measure (Fig. 6).

The increasingly equal allocation of land to land-cover types with growing uncertainties is a result of a statistical averaging effect. Our landscape-level indicators are obtained as averages formed by the area-weighted indicator levels of the individual options. Under uncertainty, the indicators of various options often show different deviations from their expected values. This leads to reduced variability when averaging the indicators for single services among land-cover types and, thus, to a buffering effect of the sometimes high uncertainties of the indicator values for the individual land-cover options. Under much enlarged uncertainty spaces, the effect of buffering uncertainties is ultimately supported by an increasingly equal allocation of land to land-cover types. In conclusion, while the high landscape diversity is required to improve multiple uncorrelated ecosystem services, high landscape diversity may also be desirable to achieve statistical averaging effects to reduce uncertainty.

### Indicator variation and achieved levels

The variability of indicator achievements is greatly reduced by the complex landscape portfolio resulting from multiple-objective optimization. This becomes obvious when comparing the indicator variability from multiple-objective optimization to that for simulated landscapes with only one land-cover type for rehabilitating the abandoned lands; see Fig. 7, which compares the diverse landscape portfolio with complete afforestation with *Alnus* or leaving all areas abandoned. For example, complete *Alnus* afforestation yields only 3 (out of 20 considered) years with positive net revenues^14^, resulting from two thinning operations and one final harvest. Thus, the temporal diversification of annual market and production risks with a homogenous *Alnus* land cover is low, leading to a high dispersion of net present values and payback periods. In the diversified landscape portfolio, this variation is effectively buffered (Fig. 7), because the pasture portfolio components are providing positive net revenues almost every year. They show much less and different patterns of variation of economic indicators compared with *Alnus*. In addition to buffering the variability of the economic consequences, the multiple-objective landscape portfolio creates a balanced and much less variable achievement of all other ecosystem services considered.

For an uncertainty level of *f*_u_=2 (Fig. 7), the worst underperformance obtained for the diversified landscape portfolio (that is, the maximum distance to the 100% achievement level) is still 73%. This guarantees an achievement level of 27% for each indicator, even under very pessimistic uncertainty scenarios. In contrast, for landscapes with single land-cover types, indicator levels of 0% are common under pessimistic uncertainty scenarios. Median achievements of indicators range from 37 to 65% for the multiple-objective landscape portfolio, while the range of median achievements is 0–100% for the single land-cover types. For examples derived under alternative uncertainty levels see Supplementary Fig. 10.

The changes in average indicator performance are small for adapting landscape portfolios to increasing uncertainty through our optimization approach (Table 3); average indicator levels decrease by 2 to 3 percentage points when considering high uncertainty levels. Only average payback periods increase notably, which reflects the growing proportion of managed lands under rising uncertainties, leading to reductions in achievement levels of 11 to 17 percentage points. Higher proportions of active rehabilitation options are needed under increasing uncertainty to buffer the considerable economic uncertainties mainly related to the afforestation options. An example for the change of the absolute indicators is contained in Supplementary Table 2.

### Sensitivity considerations

The results obtained from optimizations are quite robust under altered assumptions. For example, excluding specific groups of indicators does not usually have a big influence on the obtained landscape portfolios (Supplementary Figs 11–14), at least when uncertainty is part of the optimization. The only important influence was among the socioeconomic indicators, where the payback periods have a substantial impact on land allocation (Supplementary Fig. 13B). Disregarding the time required for recovering investments would completely remove the abandoned succession areas from the optimized land-use portfolio. Payback periods are important indicators, because access to capital is usually limited for farmers in our study area^31^.

The expected achievement levels obtained for single land-cover options influence their inclusion in or exclusion from the optimized landscape portfolios. For example, reducing the expected indicator levels for single land-cover options, while keeping those for the other options constant, leads to the exclusion of *Pinus* (uncertainty level *f*_u_=0) or low-input pasture (uncertainty levels *f*_u_=1 and *f*_u_=2, see Supplementary Fig. 15). However, when the uncertainty level is higher (*f*_u_=3), only the shares of the tested options are reduced, with all options kept in the portfolio. This illustrates a trade-off between the size of the uncertainty space and the impact of the original indicator levels. Integrating very large uncertainties may lead to the inclusion of each land-cover type into the landscape portfolio, regardless of the initial level of expected achievement. This result is intuitive, because a very large uncertainty about the performance of all options does not support strong preference for single options.

Finally, it may be argued that rarely are all ecosystem services equally important, because any discussion of indicators leads to debates about weights and preferences^32^. To simulate an increased preference for specific ecosystem services, we allocated unequal weights to the specific groups of indicators and their differences to the maximum achievement level (Supplementary Fig. 16). From this analysis, it becomes clear that preferring specific ecosystem services leads to increasingly homogenous landscape portfolios in many cases, often resulting in only one dominating land-cover type, at least when ignoring uncertainty (Supplementary Fig. 16B). Finally, if we consider uncertainties (Supplementary Fig. 16A), then the optimization suggests at least some landscape diversification, even when using very high weights for specific groups of ecosystem services. The only exception is for the indicator payback periods, for which a greatly increased preference always leads to abandoned lands not being rehabilitated (Supplementary Fig. 16).

Decision makers first have to decide whether a limited number (possibly only one) or multiple ecosystem services are of interest, and then how to weight the selected ecosystem services. Our approach can address both perspectives, with and without weighting, but we find it most convincing to consider the improvement of multiple desired ecosystem services simultaneously, which is most likely to best support a sustainable provisioning of ecosystem services at the landscape scale.

## Discussion

Our work sheds new light on the interplay of multiple ecosystem services, their uncertainties and land allocation. It shows that diverse landscape composition is advantageous from several perspectives. Considering multiple ecosystem services (multifunction effect) and integrating disturbance (uncertainty effect) both support highly diverse landscapes. However, the multifunction effect dominates compositional landscape diversity, even if only subsets of indicators are considered, as long as the indicators used are uncorrelated. If each land-cover type delivers different services, such landscape diversification appears intuitive.

Landscape diversification has been recommended elsewhere to deliver multiple services^33^,^34^ and more recently, ‘balanced' concepts have been suggested as a new paradigm for land-use^35^. The compositional diversity necessary to improve multiple services provides an implicit buffer against uncertainty so that considering additional uncertainty does not greatly change the landscape diversity. While the number of service indicators has only a minor impact on landscape composition (when considering at least eight indicators), an increasing size of the uncertainty spaces leads to increasingly equal land allocation to the land-cover types. This is due to statistical averaging effects evoked by diverse landscapes, a phenomenon called the ‘portfolio' effect^36^. However, the additional effect of uncertainty on landscape composition is small, when landscape diversification is already triggered by the multifunction effect. In light of these results, it may be justified to only consider multiple ecosystem services in land-use/cover planning, if several indicators are uncorrelated. Careful consideration is advisable, however, if indicators are correlated, because the resulting homogenized landscapes do not provide any buffer against uncertainty.

As an important result of our study, high compositional landscape diversity can reduce the often large volatility of ecosystem service indicators for individual options. While our set of indicators does not address biological diversity, our results show that complex landscapes protect a large range of ecosystem services against the adverse effects of uncertainty, a result which supports policies to prevent landscape homogenization^1^. However, our optimizations show that optimally diversified landscape portfolios that provide multiple ecosystem services are not necessarily the by-product of single-objective economic land-use decisions by risk-averse farmers. Furthermore, our research does not support a win–win scenario between optimization of ecological and socioeconomic indicators (Fig. 5). In addition, divergent land allocation under uncertainty becomes clear, if we consider results obtained from optimizing only single economic objectives (*cf* Supplementary Figs 6 and 7). Risk-averse farmers will probably diversify their land-uses and provide substantially more ecosystem services to society, compared with risk-neutral farmers, but their objective to diversify economic risks is likely to lead to different patterns of land allocation. The economically diversified landscapes may largely exclude succession areas (areas left abandoned; Supplementary Fig. 6), which also provide important ecosystem services and biodiversity^37^. Since economically optimal diversification is driven by the desire to reduce uncertainties, access to insurance or specific agricultural policies to reduce uncertainties will counteract diversification^8^,^38^,^39^. Consequently, the levels of ecosystem services will decrease with the reduced economic uncertainties for farming. In summary, while our study does not support a general win–win between optimizations under either ecological or socioeconomic objectives, it appears notable that the optimization results converge, if they address the uncertainties of several ecosystem service indicators. Therefore, we may minimize trade-offs, if both ecological and socioeconomic perspectives consider uncertainties.

It remains a complex scientific task to integrate ecosystem services into landscape management^40^, particularly if uncertainties about ecosystem services prevail. Although promising analytical approaches that use restoration scenarios exist^41^, such as regional land-use scenarios^42^,^43^ or national level land-use optimization based on economic valuation of ecosystem services^44^, a combination with programming-based multiple-objective optimization at the landscape scale is still largely lacking^45^ (see Estrella *et al*.^19^ and Chang *et al*.^46^ for examples). Moreover, approaches to consider uncertainties of multiple ecosystem services are rare.

Our approach to uncertainty is a type of robust optimization^47^, which secures improved levels for multiple ecosystem services based on sets of possible indicator values, derived from uncertainty spaces. However, we have not directly addressed the opportunity to adapt the landscape portfolio according to changes in the ecosystem indicators, for example under changing environmental or market conditions. More adaptive approaches to uncertainty would incorporate the learning about the effectiveness of the different options for achieving different objectives into the planning solution itself^48^. Adaptive governance assumes that landscapes need to be understood and governed as complex social–ecological systems rather than as ecosystems alone^49^. Mechanistic modelling approaches, as suggested by our study, have a high potential to inform an iterative decision-making process as outlined by Polasky *et al*.^50^. In response to temporal changes in the expected ecosystem service levels for the considered options, optimizations can be rerun and revised landscape portfolios may be offered. However, adaptation options may be limited from a financial point of view, given the quite long payback periods (10 to 32 years). A rapid and early change in the landscape portfolio will thus often only be justified when severe changes are expected or completely new and deviating knowledge arises. However, a diversified portfolio offers more convenient options for adaptation compared to a more specialized landscape.

Further approaches that have been proposed to manage uncertainty in ecosystems are scenario planning^51^,^52^ and resilience assessment^4^,^53^. The success of using scenario planning for decisions under uncertainty strongly depends on the range of expert opinions, stakeholder objectives and hence the identified scenarios. Scenario planning does not assign probabilities to different alternative landscape pathways and uncertainty is usually reflected by the selected scenarios themselves^54^. Some authors couple scenario analysis with stochastic simulations to test the robustness of political strategies under changing levels of uncertainty^55^,^56^,^57^. Resilience thinking is strongly interconnected with adaptive management and highlights the importance of considering the interdependencies of social and biophysical systems. Fischer *et al*.^58^ have encouraged embedding optimization approaches into a resilience thinking framework. In line with this, our robust multiobjective optimization can offer a richer understanding of the complex dynamics of rehabilitation and landscape planning, which can be used for and expanded by participatory and interdisciplinary approaches adopted in scenario planning and resilience thinking.

Alternatives to indicator based multiple-objective optimization under uncertainty include spatially explicit economic optimization based on monetary value coefficients. For example, Bateman *et al*.^44^ offer important advances using this approach and conclude that targeted planning and optimization of both ecosystem services and agricultural market values is needed. Using normalized ecosystem service indicators for different land-use options, instead of assessing changes in their monetary value, has significant advantages for safeguarding ecosystem services. In this respect our model avoids the degradation of important services. For instance, diminishing or even losing a service, such as water filtration and retention, because another service achieves higher economic value coefficients, can have far-reaching consequences for human wellbeing that might be difficult to enumerate in monetary terms.

There remain some challenges for optimizing multiple ecosystem services at the landscape scale. For example, there will always be a debate about the importance of indicators^32^, including which indicators are essential and which are not. In our study region, soil erosion can be an important aspect of land management, but is not yet directly addressed by our set of indicators. However, given the large number of indicators used and the robustness of our results, we do not believe that more indicators would substantially change the results (Fig. 4). Moreover, considering uncertainty of ecosystem indicators effectively protects against unbalanced decision-making, should we have evaluated one option too positively. For our study, one could argue that we may have been too optimistic in evaluating the impact of afforestation with *Pinus* on soil quality and carbon stocks, compared, for example, with results obtained by Hall *et al*.^59^ or Henry *et al*.^60^. However, *Pinus* never dominates our land-use portfolios, so we may conclude that our approach provides a good buffer against possible bias.

One aspect excluded from our study is the impact of landscape configuration on ecosystem services and optimization of landscape diversity. Configurational landscape heterogeneity (for example, size and arrangement of patches) was less important as a filter of diversity for arthropod communities compared with compositional landscape heterogeneity^1^. Considering landscape configuration may be quite important to cover spill-over effects^61^ as well as synergistic or antagonistic effects between mixed crops (see Neuner *et al*.^62^ for the case of mixed-species forests). However, the balanced composition of land-cover types that we derived leaves enough space to optimize landscape configuration^63^. For example, our intense pasture option may well be embedded into an advantageous landscape configuration together with options that provide strong regulating services, such as afforestation with native trees. This can reduce adverse spill-over effects, for example, those resulting from fertilizer use. Thus, possible impacts and feedback of ecosystem services controlled by landscape configuration on optimal landscape diversity forms a field for further future research.

Our new optimization approach could inform the actual land-use planning in our study region. Here abandoned pastures cover 35% of the total pasture area^64^. Among the reasons for the abandonment of these pastures is the invasion of weeds—mainly tropical bracken. Bracken is resistant to fire—the most common local tool to control weeds^65^. However, long-running experiments show that these areas may actually be rehabilitated by afforestation or repasturization^14^. The state of Ecuador has enacted a national plan of forest restoration^66^, which identifies a total area of 1,599,342 ha, mainly consisting of degraded/abandoned pastures or other agricultural lands, as suitable for restoration/rehabilitation. The national plan considers priority areas for aspects such as the protection of water resources, biodiversity or degraded sites inside protected areas. The government has declared the restoration/rehabilitation of 300,000 ha as an official target and aims at restoring 500,000 ha in total by 2017 (ref. 66). The objectives of restoration/rehabilitation are included in developmental programmes on regional and local scales^67^. However, their scope is limited to mainly afforestation options or leaving areas abandoned, while whole-landscape portfolios and their quantitative impacts on ecosystem services are not considered.

The practice of landscape planning in Ecuador described above may be improved by our optimization approach, which delivers information on the land needed for several land-cover types in the form of an optimized landscape composition for rehabilitation areas^63^. Combined with rule sets to make the intended distribution of land-cover types spatially explicit, priority zones may be formed (Supplementary Fig. 17). Considering these landscape zones may help to allocate payments or other incentives to landowners to stimulate rehabilitation (Supplementary Methods).

In conclusion, our study supports understanding of mechanisms behind the complex interplay between multiple ecosystem services, their uncertainties and the optimal allocation of land. Moreover, the method we have developed allows for a more differentiated consideration of positive externalities incurred by risk-averse farmers through their desire to mitigate future economic uncertainties. It ultimately provides strong support for a more complex landscape composition, addressing multiple and uncertain ecosystem services.

## Methods

### Data and land-cover options

This study builds on the indicators published in a synthesis paper^14^, which summarizes the results of a multi-disciplinary research initiative focused on ecosystem studies in southern Ecuador, since 1998. In that paper, models parameterized with the field data served to obtain the results for some ecosystem service indicators over a 20-year period assuming nearly constant environmental conditions (Table 1). Other indicators were either measured directly in the field or obtained from interviews (see Knoke *et al*.^14^ for details). Supplementary Methods contain a justification of the indicators used.

We refer to the following land-cover options investigated in the context of rehabilitation of abandoned tropical pastures: (I) to leave areas abandoned (resulting in succession areas, referred to as ‘abandoned'); (II) afforestation with the native tree species *Alnus acuminata* (*Alnus*); (III) the exotic *Pinus patula* (*Pinus*); (IV) converting to low-input pasture with subsequent low-input management (low-input pasture); or (V) converting to intense pasture with subsequent intense management (intense pasture).

### Uncertainty and the optimization of multiple services

**Figure 3:**
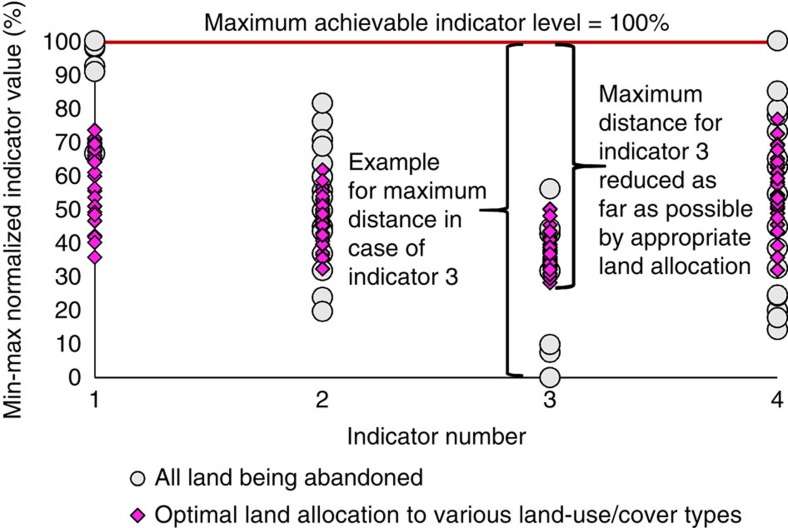
Schematic description illustrating improvement of achieved minimum levels of indicators. The graph shows the min–max normalized (that is, indexed) achievement levels (*y* axis) for four potential indicators (*x* axis). The indexed indicators are scaled from zero (least desirable indicator value) to 100% (most desirable value). The four indicators given as examples represent the pH (indicator 1), soil organic carbon (indicator 2), base saturation (indicator 3) and carbon in microbial biomass (indicator 4). Each point represents the indicator achievement level for the considered uncertainty scenarios (2^5^=32) for each indicator. Results for two land-use allocation scenarios are depicted: grey circles represent the indicator values under the scenario that all of the land is allocated to abandoned land. For indicator number 3, abandoned land would give the least desirable indicator value of zero for some of the uncertainty scenarios. Hence, the maximum distance to the most desirable value is 100%. Pink diamonds represent the indicator achievement levels under the ‘optimized' scenario, consisting of a mixture of various land-cover types, which buffers against uncertainty. In this scenario, land is allocated to the five land-cover types (four rehabilitation options plus the ‘abandoned' category) in a way that minimizes the maximum distance to the 100% achievement level. Here landscape-level indicators consist of the sum of the indicators recorded for the individual land-cover types (reported in Table 1), which have been multiplied with the area fraction allocated to the corresponding land-cover type.

**Figure 4:**
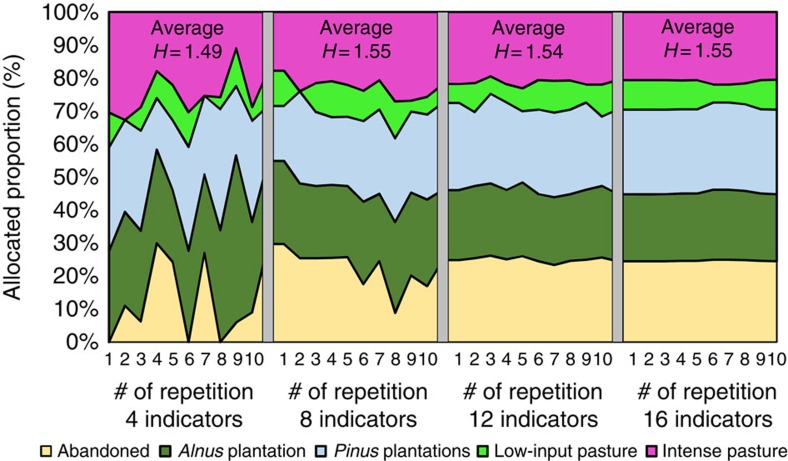
Impact of the number of indicators considered (multifunction effect). Obtained composition of the landscape portfolios is relatively stable when using eight indicators or more. On the basis of four randomly selected indicators the average landscape composition is slightly less complex compared with that of landscapes that result from considering eight or more indicators. Random experiments with 10 repetitions with different indicator combinations for each size of the indicator set (4, 8, 12, and 16 indicators, all optimizations with uncertainty level *f*_U_=2.0) were carried out, with half of the indicators drawn from the ecological and half from the socioeconomic set. *H* stands for Shannon's index computed for the average landscape composition.

Our new method starts with integrating non-stochastic uncertainty sets (Fig. 2 and Knoke *et al*.^17^) for each of the 22 indicators into the optimization. This allows for considering the uncertainty of ecosystem service indicators in a robust fashion (see Ben-Tal *et al*.^47^ for a comprehensive justification of robust optimization). For various sizes of these uncertainty sets, we integrate all possible combinations of maximum optimistic and pessimistic deviations for each indicator and land-cover type, resulting in 32 uncertainty scenarios for each indicator. By appropriately allocating proportions of abandoned pasture lands, *a*_l_, to five specific land-cover types, *i*, we then minimize the difference between the (hypothetically) achievable indicator level (100%) and the actually achieved level for each of the 22 ecosystem service indicators at the landscape scale (Fig. 3). The ecosystem service indicators, *R*_iu_, are computed at a landscape level as area-weighted averages^19^,^46^ based on the indicator values achieved by the single land-cover options under each uncertainty scenario (22 indicators by 32 uncertainty scenarios gives 704 scenarios altogether). During the optimization process a set of indicator values, *R*_liu_, for each single land-cover option form our input parameters and the proportion of land allocated to each of the five land-cover options form the variables to be optimized (decision variables). The outcome is an optimum allocation of area proportions to the land-cover types considered for rehabilitation. To summarize, we use a variant of goal programming^18^,^68^, also referred to as compromise programming^19^,^23^,^46^, to minimize non-achievement of maximum ecosystem service levels considering indicator variation by means of inclusive uncertainty sets^17^.

To implement our method we impose one achievement function per indicator and uncertainty scenario (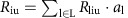), which controls the absolute achievement level for each ecosystem service under each uncertainty deviation at the landscape scale. That means that all uncertainty scenarios, covering various combinations of optimistic and pessimistic parameters for each indicator and each land-cover option, have their own achievement function. We represent uncertainty by the standard error of the mean (s.e.m._li_) in our data sets^14^ (Table 1). To integrate uncertainty into the optimization, we refer to positive (optimistic) or negative (pessimistic) deviations, *u*_i_, from the recorded indicator. Considering only two land-cover options, the optimistic and pessimistic indicator levels form rectangular uncertainty areas with four corner points (one for each uncertainty scenario, see example in Fig. 2). The actual method, however, considers five-dimensional uncertainty spaces with 32 corner points, to integrate optimistic and pessimistic indicator levels in all possible combinations for all five land-cover options (2^5^=32 uncertainty scenarios). To explore the impact of increasing uncertainty on the resulting landscape structure, we sequentially increase the size of the uncertainty spaces, as is schematically shown for the simple case of two land-cover options in Fig. 2.

All landscape-level indicators obtained, *R*_iu_, have been scaled between zero (least desirable) and 100% (most desirable) to form indexed values^14^,^19^,^23^,^46^,^69^, *p*_iu_. The scaling was achieved by taking the difference of each landscape-level indicator value and the least desirable indicator value and dividing this difference by the range of the indicator among the options considered (most desired minus least desired, equation (1)). The resulting quotient multiplied by 100 gives the relative position between the most and the least desired values of the indicator achieved at the landscape scale. Finally, we imposed one unitary constraint, *D*_u_, on each indicator, *i*, and uncertainty scenario, *u*. In total, we considered 704 constrained achievement functions for 22 indicators, each represented with 32 uncertainty scenarios. The constraints control the maximum distance to the highest achievement level; we seek to minimize this distance to reduce underperformance to lowest possible level. This distance depends on the allocation of land proportions, *a*_l_, to the five land-cover types. We minimized the tolerated distance iteratively, by reducing *D*_u_, as long as none of the constraints were violated, to find *D*_u,min_. Frontline Solvers V2015 (15.0.2.0) was used with the Standard Linear Programming Engine to solve our constraint-based formulation of our problem. We summarize the applied method as follows:


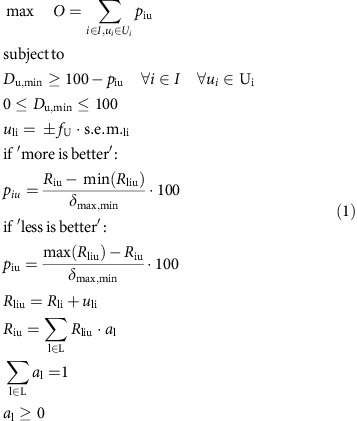


with


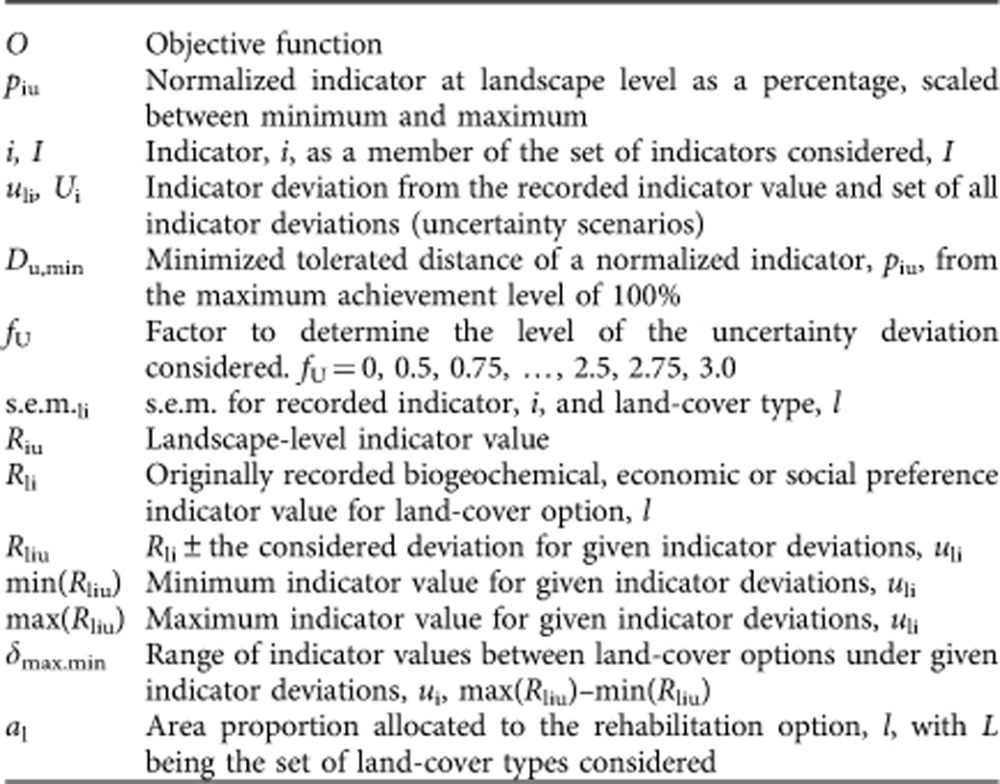


We used the same model for the single-objective optimization, but reduced the optimization by only considering one indicator and its 32 uncertainty scenarios at the same time.

**Figure 5:**
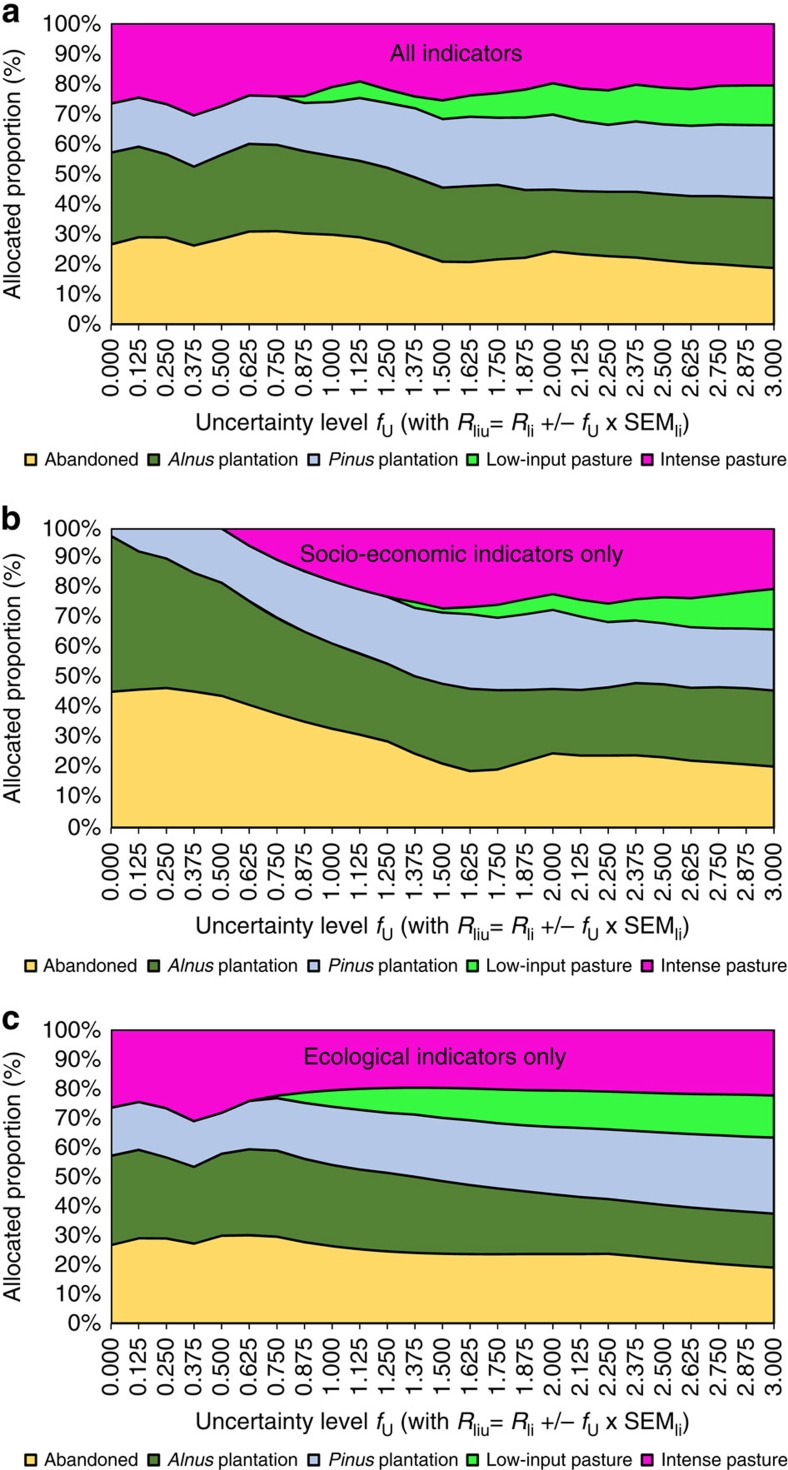
Impact of the uncertainty effect. The proportions of land allocated to the land-cover types do not heavily depend on the modelled level of uncertainty when (**a**) all indicators or (**c**) only ecological indicators are used. Uncertainty has a greater impact on landscape composition when (**b**) only the socioeconomic indicators are considered (indicators that are often correlated). Uncertainty level refers to multiples of the s.e.m. used to increase the size of the uncertainty boxes, as displayed in Fig. 2. Subscripts for indicator value, *R*: *l* for land-use option, *i* for indicator, and *u* for uncertainty deviation from recorded indicator. s.e.m._li_ of *R* for land-use options, *l*, and indicators, *i.*

**Figure 6:**
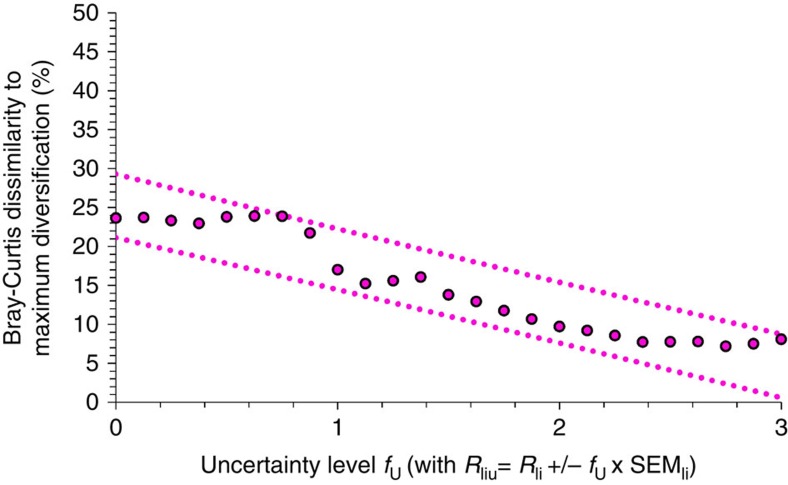
Dissimilarity of landscape portfolios. The Bray–Curtis index expresses the dissimilarity of the optimized landscape portfolios to a theoretically maximally diversified landscape (that is, coverage of 20% for each land-cover type, with a maximum Shannon index of land-cover diversity of 1.61). Minimum dissimilarity is 0%, maximum dissimilarity is 100%. Dotted lines refer to 95% confidence limits for single dissimilarity values. Subscripts for indicator value, *R*, *l* for land-use option, *i* for indicator, and *u* for uncertainty deviation from recorded indicator. s.e.m._li_ of *R* for land-use option, *l*, and indicator, *i*.

**Figure 7:**
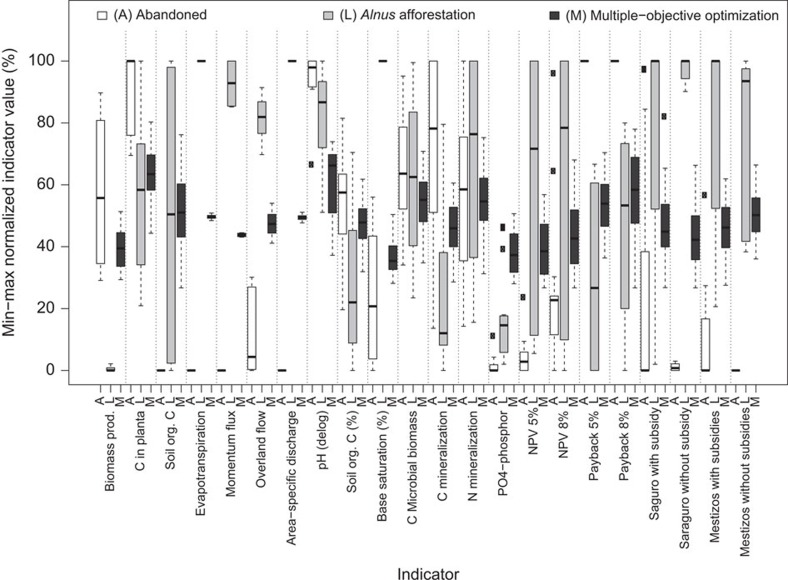
Normalized landscape scale indicators. Whiskers: minima and maxima, boxes formed by quartiles comprising the median. Multiple-objective optimization is compared with single land-use options for an uncertainty level of *f*_u_=2.

The multidimensional uncertainty spaces for the five land-cover types may form a rough surface with 32 corner points. Altering the size of the uncertainty spaces changes their shape. This explains the slight noise in the composition of land-cover portfolios when altering the level of uncertainty and with it the shape of the uncertainty space (for example, Fig. 5a,b). However, this noise had no influence on the validity of the basic results.

Estrella *et al*.^19^ have published an innovative approach to optimal land allocation with the aim of achieving multiple ecosystem services, which shows some similarities to our approach. While integrating uncertainties was not the aim of their study, the authors used an alternative to our constraint-based formulation of the optimization problem (equation (1)). Their approach minimized maximum non-achievement (*D*_u,min_) directly, using an appropriate objective function (see Supplementary Methods for mathematical formulation). The authors averaged the objective of minimizing maximum non-achievement, which is a non-compensatory approach, with another objective, which allows for compensatory minimization of distances (see also Eyvindson and Kangas^23^). Compensatory minimization allows high performance in one indicator to compensate for low performance in another indicator. Here the part of the objective function that minimizes maximum non-achievement is relevant to our study, because it follows a similar idea as our approach. In contrast to Estrella's *et al*.^19^ approach, we used a constraint-based formulation referring to robust optimization^17^,^47^ for addressing uncertainty, which is excluded in Estrella *et al*.^19^.

### Landscape composition

Shannon's index^70^ (represented by *H* in equation (2)) has been computed for landscape portfolios, with *a*_l_ being the decimal proportion of land allocated to each land-cover option in a given portfolio.


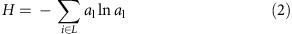


Bray–Curtis measure of dissimilarity (BC_ur_in equation (3)) has been computed based on the relative proportions (*p*_l_ in per cent) of the land allocated to each land-cover type (*l*). The landscape portfolio obtained under a theoretical maximum of diversification (each land-cover-type comprising 20% of the available land) has been used as a reference (index *r*). The proportions of each land-cover type achieved in landscape portfolios under increasing levels of uncertainty (index *u*) were compared with those for the reference landscape portfolio.


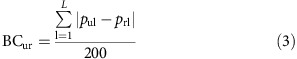


A BC_ur_ close to zero means low dissimilarity, while a BC_ur_ close to 100% indicates high dissimilarity.

### Data availability

The authors confirm that all the relevant data are available through the lead author.

## Additional information

**How to cite this article:** Knoke, T. *et al*. Compositional diversity of rehabilitated tropical lands supports multiple ecosystem services and buffers uncertainties. *Nat. Commun.* 7:11877 doi: 10.1038/ncomms11877 (2016).

## Supplementary Material

Supplementary Information Supplementary Figures 1-17, Supplementary Tables 1-4, Supplementary Methods and Supplementary References.

## Figures and Tables

**Table 1:** Considered indicators and their uncertainties, adopted from Knoke *et al*. 14 (to quantify possible deviations from recorded indicator levels we use the s.e.m._li_, for each land-cover type, *l*, and indicator, *i*, given in parentheses).

Indicator values							
Indicator group	Indicator	Unit	Abandoned	*Alnus* plantation	*Pinus* plantation	Low-input pasture	Intense pasture
Carbon relationships	Biomass production	mg ha^−1^ per year	31.8 (±4.8)	7.7 (±0.6)	8.9 (±0.4)	26.5 (±4.4)	50.0 (±2.3)
	Carbon in planta	Mg ha^−1^	33.0 (±2.9)	24.5 (±2.3)	29.6 (±1.4)	12.5 (±1.2)	25.8 (±3.4)
	Soil organic carbon	Mg ha^−1^	87.3 (±5.3)	91.7 (±6.8)	93.5 (±4.6)	91.8 (±4.9)	96.3 (±5.1)
							
Climate regulation	Evapotranspiration	mm per year	928 (±3.8)	1,597 (±4.1)	1,410 (±1.1)	1,186 (±5.8)	1,167 (±5.1)
	Momentum flux	kg m^−1^ s^−2^	0.018 (±0.00028)	0.285 (±0.0156)	0.294 (±0.00038)	0.023 (±0.00003)	0.026 (±0.0004)
							
Hydrological regulation	Overland flow	mm per year	75 (±3.7)	38 (±0.8)	29 (±1.5)	75 (±2.8)	77 (±2.9)
	Area-specific discharge	mm per year	927 (±6.9)	283 (±4.0)	471 (±2.7)	677 (±7.0)	695 (±6.1)
							
Soil quality	pH (delog used for optimization)		4.50 (±0.09)	4.30 (±0.04)	3.60 (±0.13)	4.50 (±0.18)	4.10 (±0.09)
	Soil organic carbon (SOC)	%	10 (±0.2)	8 (±0.7)	7 (±0.8)	11 (±0.6)	12 (±0.4)
	Base saturation	%	12 (±2.6)	30 (±1.8)	6 (±1.2)	17 (±1.3)	12 (±1.3)
	Carbon in microbial biomass	mg kg^−1^	1,088 (±51)	1,065 (±80)	576 (±75)	1,065 (±102)	1,359 (±65)
	Carbon mineralization	g CO_2_–C per kg SOC	4 (±0.18)	3 (±0.13)	4 (±0.49)	4 (±0.31)	3 (±0.27)
	Nitrogen mineralization	mg N per kg per day	2 (±0.27)	3 (±0.49)	2 (±0.31)	1 (±0.22)	3 (±0.12)
	PO_4_–Phosphor	mg kg^−1^	1 (±0.09)	1 (±0.22)	6 (±1.21)	1 (±0.13)	6 (±1.79)
							
Net present value	At 5% discount rate	US$ per ha	0 (±0)	1,435 (±649)	1,322 (±586)	127 (±146)	1,060 (±234)
	At 8% discount rate		0 (±0)	619 (±394)	561 (±373)	-156 (±129)	485 (±234)
							
Payback period	At 5% discount rate	years	0 (±0)	16 (±3)	16 (±3)	18 (±6)	10 (±2)
	At 8% discount rate		0 (±0)	16 (±4)	16 (±4)	32 (±4)	13 (±4)
							
Preference Saraguros	With subsidy	Answers with preference rank 1 or 2	4 (±2)	14 (±3)	12 (±3)	5 (±2)	4 (±2)
	Without subsidy		0 (±0)	19 (±3)	9 (±3)	3 (±2)	8 (±3)
							
Preference Mestizos	With subsidy		5 (±2)	19 (±4)	15 (±3)	12 (±3)	12 (±3)
	Without subsidy		0 (±0)	16 (±3)	17 (±4)	14 (±3)	10 (±3)

**Table 2:** Changes in aggregated indicators when including multiple ecosystem services: Achieved average min–max normalized indicators after optimization with increasing numbers of indicators.

Achieved average min–max normalized indicators in % for optimization with *n* indicators					
Indicator group according to Table 1	*n*=4	8	12	16	Change
Carbon relationships	55	55	55	55	Constant
Climate regulation	59	50	48	48	Decrease
Hydrological regulation	56	48	46	47	Decrease
Soil quality	53	53	53	53	Constant
Net present value	76	66	64	64	Decrease
Payback period	42	48	50	50	Increase
Preference Saraguros	53	45	42	42	Decrease
Preference Mestizos	70	61	58	59	Decrease

**Table 3:** Changes for adapting to uncertainty: achieved average min–max normalized indicators after optimization with various indicator sets for increasing levels of uncertainty (changes of ±2% were denoted as ‘constant').

Achieved average min–max normalized indicators in % for uncertainty level *f*_u_						
Optimization with	Indicator group according to Table 1	*f_u_*=0	1	2	3	Change
**All indicators**	Carbon relationships	57	54	54	53	Decrease
	Climate regulation	49	46	48	50	Constant
	Hydrological regulation	47	45	46	49	Constant
	Soil quality	56	55	52	52	Decrease
	Net present value	69	62	62	64	Decrease
	Payback period	55	54	48	44	Decrease
	Preference Saraguros	46	43	42	45	Constant
	Preference Mestizos	58	56	59	63	Increase
						
**Socioeconomic**	Carbon relationships	44	54	55	52	Increase
**indicators**	Climate regulation	53	49	49	49	Decrease
	Hydrological regulation	50	48	48	47	Decrease
	Soil quality	55	54	53	53	Constant
	Net present value	59	65	66	63	Increase
	Payback period	62	57	51	45	Decrease
	Preference Saraguros	54	45	44	44	Decrease
	Preference Mestizos	53	55	59	62	Increase
						
**Ecological**	Carbon relationships	57	54	54	54	Decrease
**indicators**	Climate regulation	49	49	46	48	Constant
	Hydrological regulation	47	47	45	46	Constant
	Soil quality	56	54	53	52	Decrease
	Net present value	69	65	61	62	Decrease
	Payback period	55	51	48	44	Decrease
	Preference Saraguros	46	45	41	41	Decrease
	Preference Mestizos	58	59	59	62	Increase
