# Supplementary material for: Compositional diversity of rehabilitated tropical lands supports multiple ecosystem services and buffers uncertainties
Source: Nat Commun. 2016 Jun 13;7:11877. doi: 10.1038/ncomms11877 (PMC4910018; doi:10.1038/ncomms11877)
Supplement: Supplementary Information — Supplementary Figures 1-17, Supplementary Tables 1-4, Supplementary Methods and Supplementary References. [file ncomms11877-s1.pdf]

Supplementary Figures

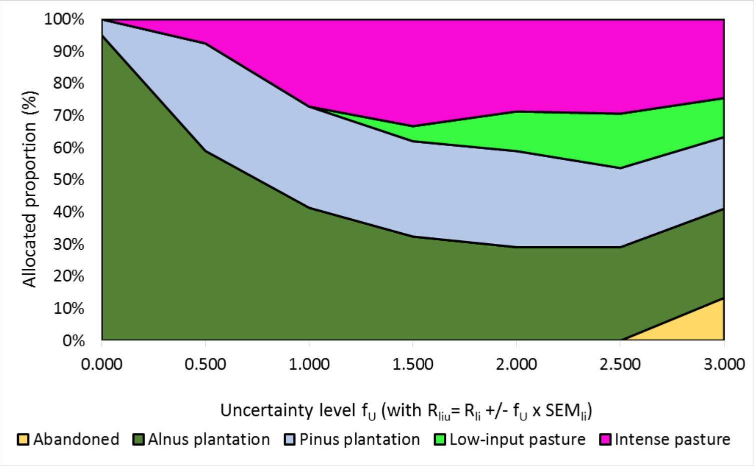

Supplementary Figure 1. Land allocation with seven highly correlated indicators (Pearson correlation > 0.7): Evapotranspiration, momentum flux, net present value with discount rates 5% and 8%, preference of Saraguros with and without subsidies, preference of Mestizos with subsidies. All indicators show bilateral correlation of more than 0.7. Using only these indicators, almost all land is allocated to *Alnus* under zero uncertainty. Leaving area abandoned plays no role over almost the whole range of considered uncertainty levels. The maximum distance to the 100% achievement level was minimized for the seven indicators each with 32 uncertainty scenarios, considered at each uncertainty level 0.00 x SEM_l, 0.25 x SEM_l, …, 2.75 x SEM_l, 3.00 x SEM_l, with SEM_l being the standard error of the estimate for a land-cover option, *l*.

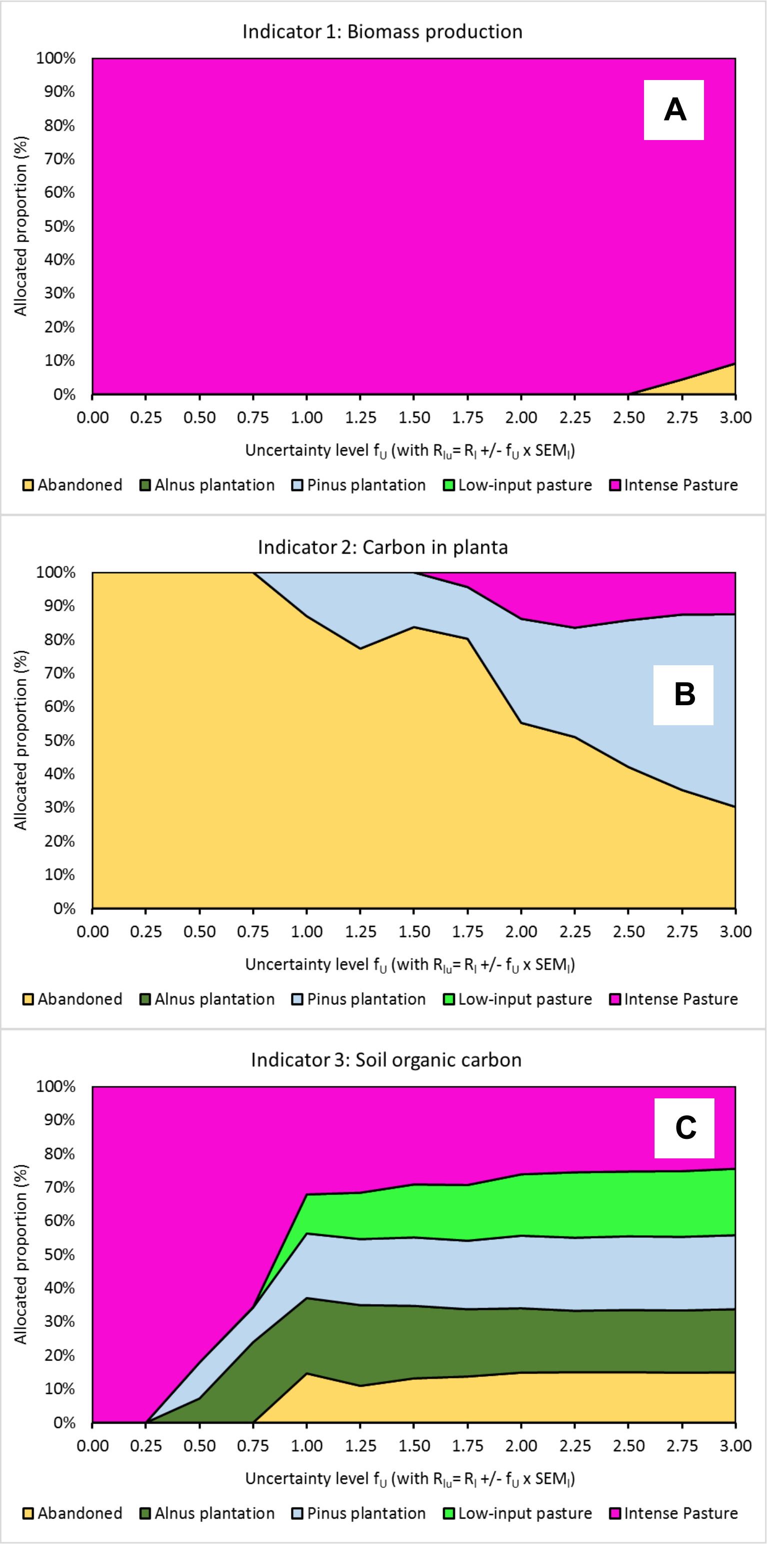

Supplementary Figure 2. Indicators for **Carbon relationships**[1] used for single objective optimization. The maximum distance to the 100% achievement level was minimized for 32 uncertainty scenarios considered at each uncertainty level 0.00 x SEM_l, 0.25 x SEM_l, …, 2.75 x SEM_l, 3.00 x SEM_l, with SEM_l being the standard error of the estimate for a land-cover option, *l*.

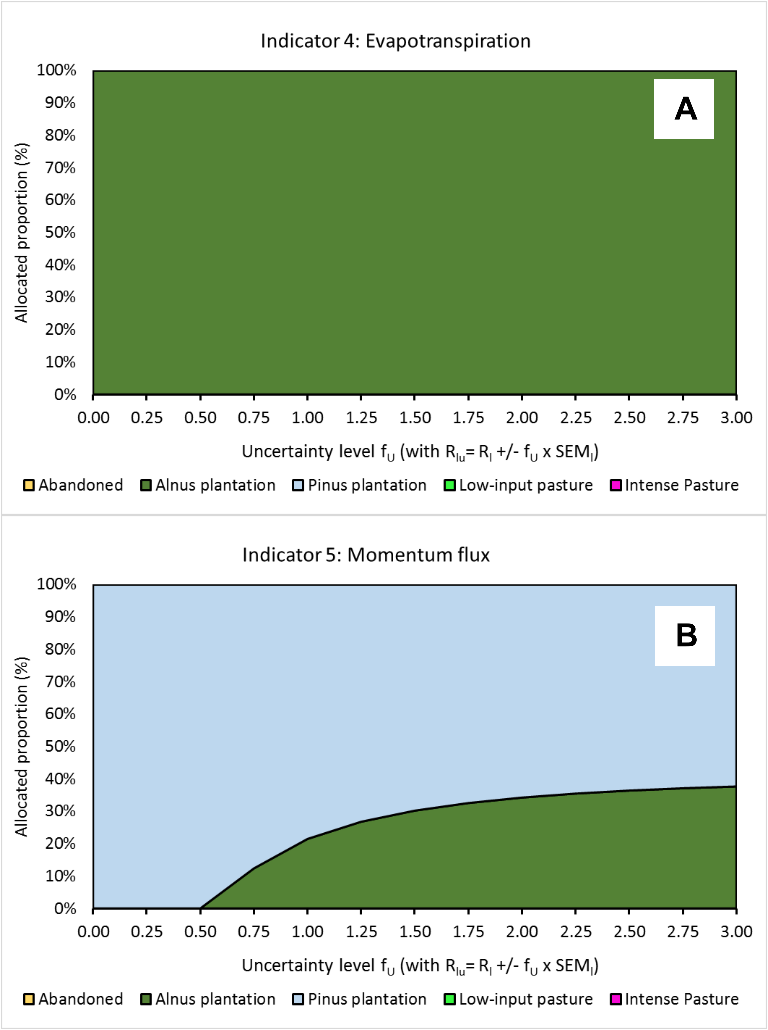

Supplementary Figure 3. Indicators for **Climate regulation**[1] used for single objective optimization. The maximum distance to the 100% achievement level was minimized for 32 uncertainty scenarios considered at each uncertainty level 0.00 x SEM_l, 0.25 x SEM_l, …, 2.75 x SEM_l, 3.00 x SEM_l, with SEM_l being the standard error of the estimate for a land-cover option, *l*. When only one colour appears (A), the uncertainty considered was too small to suggest diversification.

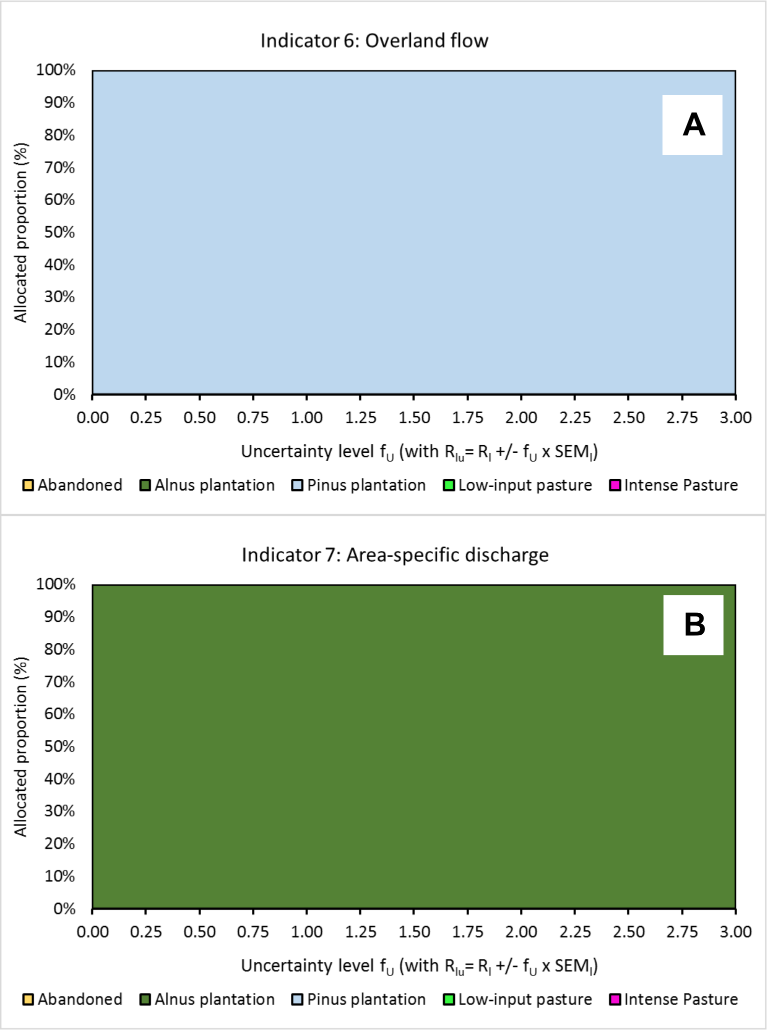

Supplementary Figure 4. Indicators for **Hydrological regulation**[1] used for single objective optimization. The maximum distance to the 100% achievement level was minimized for 32 uncertainty scenarios considered at each uncertainty level 0.00 x SEM_l, 0.25 x SEM_l, …, 2.75 x SEM_l, 3.00 x SEM_l, with SEM_l being the standard error of the estimate for a land-cover option, *l*. When only one colour appears (A, B), the uncertainty considered was too small to suggest diversification.

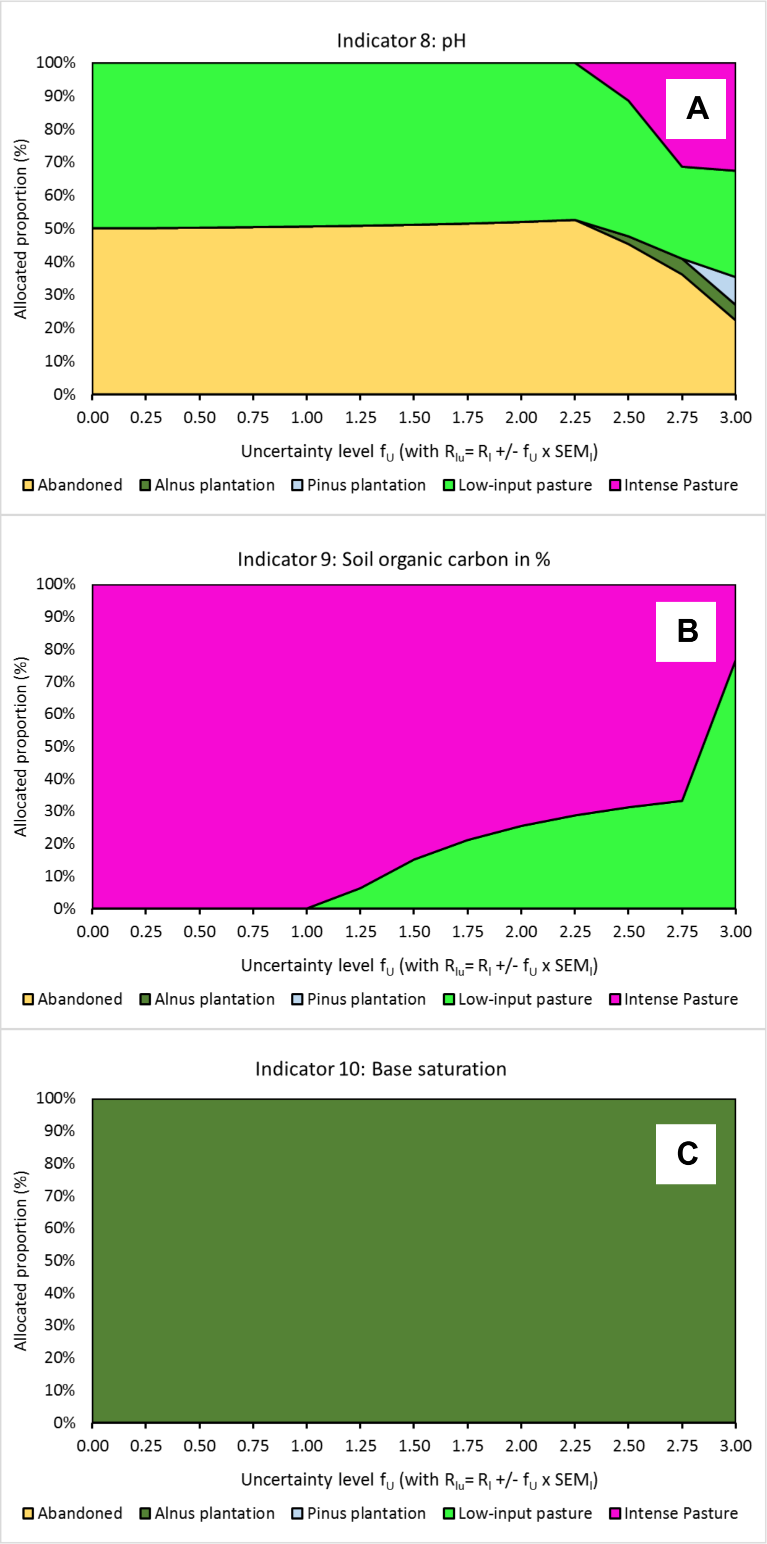

Supplementary Figure 5. Indicators for **Soil quality**[1] used for single objective optimization. The maximum distance to the 100% achievement level was minimized for 32 uncertainty scenarios considered at each uncertainty level 0.00 x SEM_l, 0.25 x SEM_l, …, 2.75 x SEM_l, 3.00 x SEM_l, with SEM_l being the standard error of the estimate for a land-cover option, *l*. When only one colour appears (C), the uncertainty considered was too small to suggest diversification.

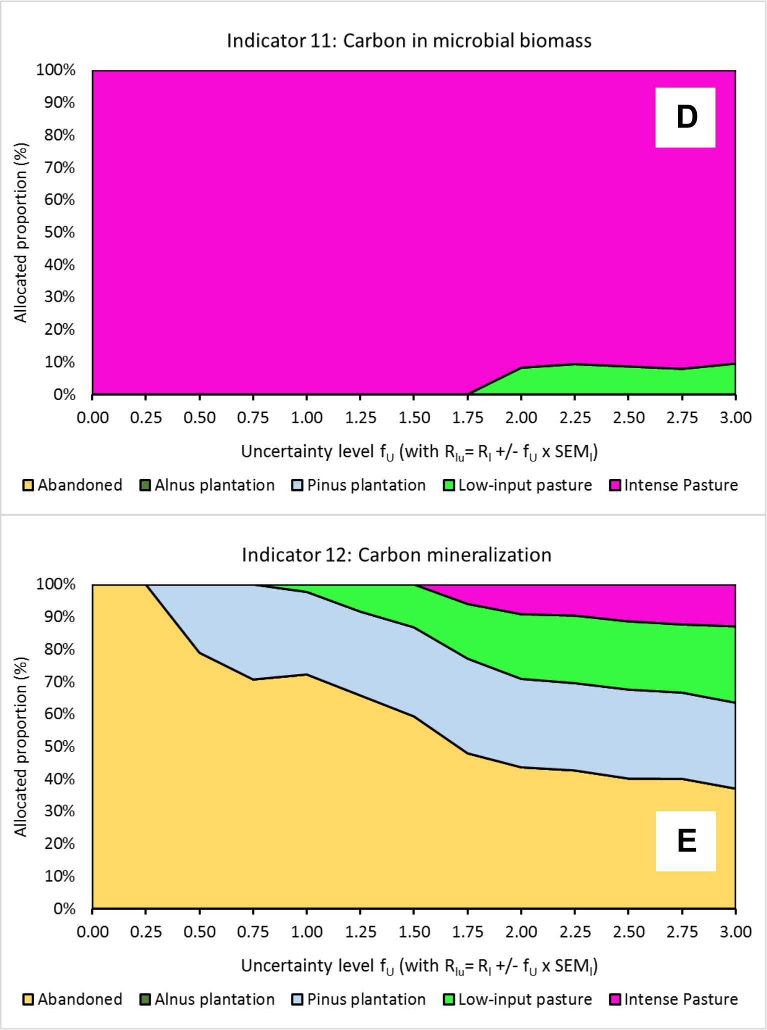

Supplementary Figure 5 continued. Indicators for **Soil quality**[1] used for single objective optimization. The maximum distance to the 100% achievement level was minimized for 32 uncertainty scenarios considered at each uncertainty level 0.00 x SEM_l, 0.25 x SEM_l, …, 2.75 x SEM_l, 3.00 x SEM_l, with SEM_l being the standard error of the estimate for a land-cover option, *l*.

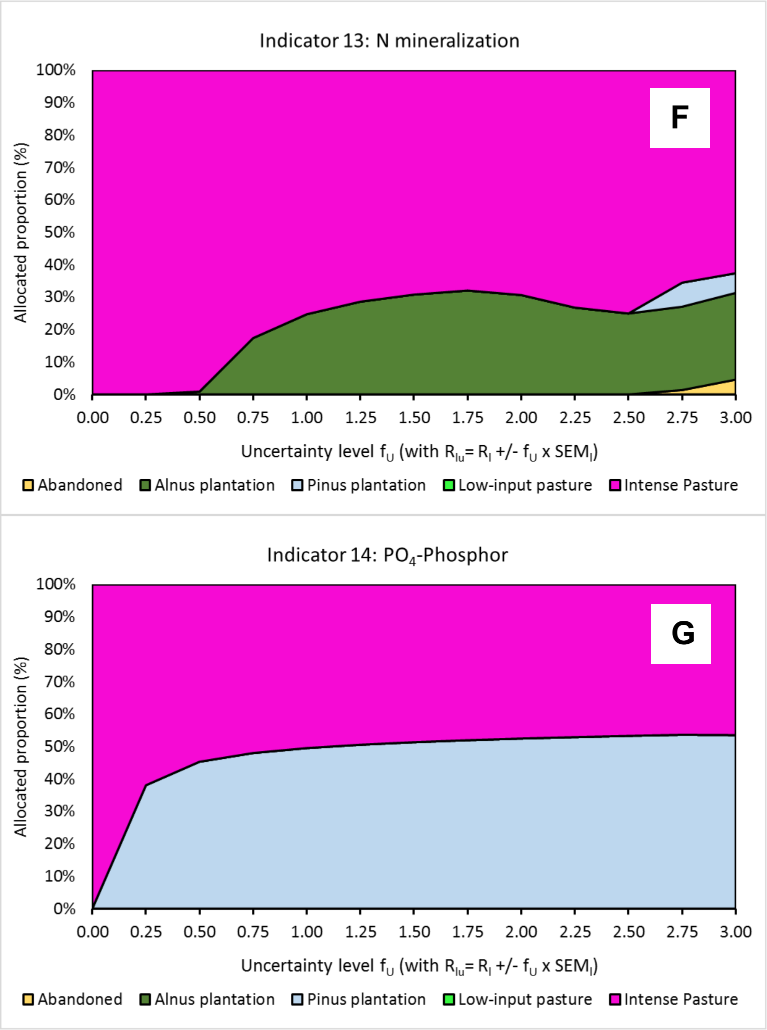

Supplementary Figure 5 continued. Indicators for **Soil quality**[1] used for single objective optimization. The maximum distance to the 100% achievement level was minimized for 32 uncertainty scenarios considered at each uncertainty level 0.00 x SEM_l, 0.25 x SEM_l, …, 2.75 x SEM_l, 3.00 x SEM_l, with SEM_l being the standard error of the estimate for a land-cover option, *l*.

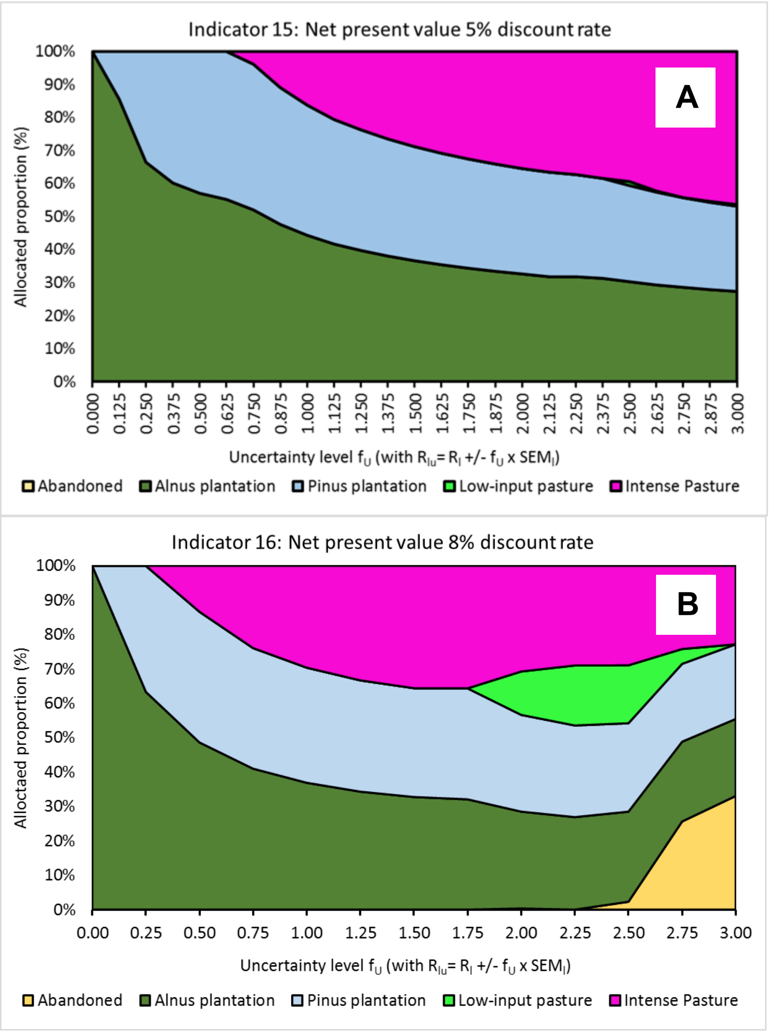

Supplementary Figure 6. Indicators for **Net present values[1]** (sum of discounted net revenues) used for single objective optimization. The maximum distance to the 100% achievement level was minimized for 32 uncertainty scenarios considered at each uncertainty level 0.00 x SEM_l, 0.25 x SEM_l, …, 2.75 x SEM_l, 3.00 x SEM_l, with SEM_l being the standard error of the estimate for a land-cover option, *l*.

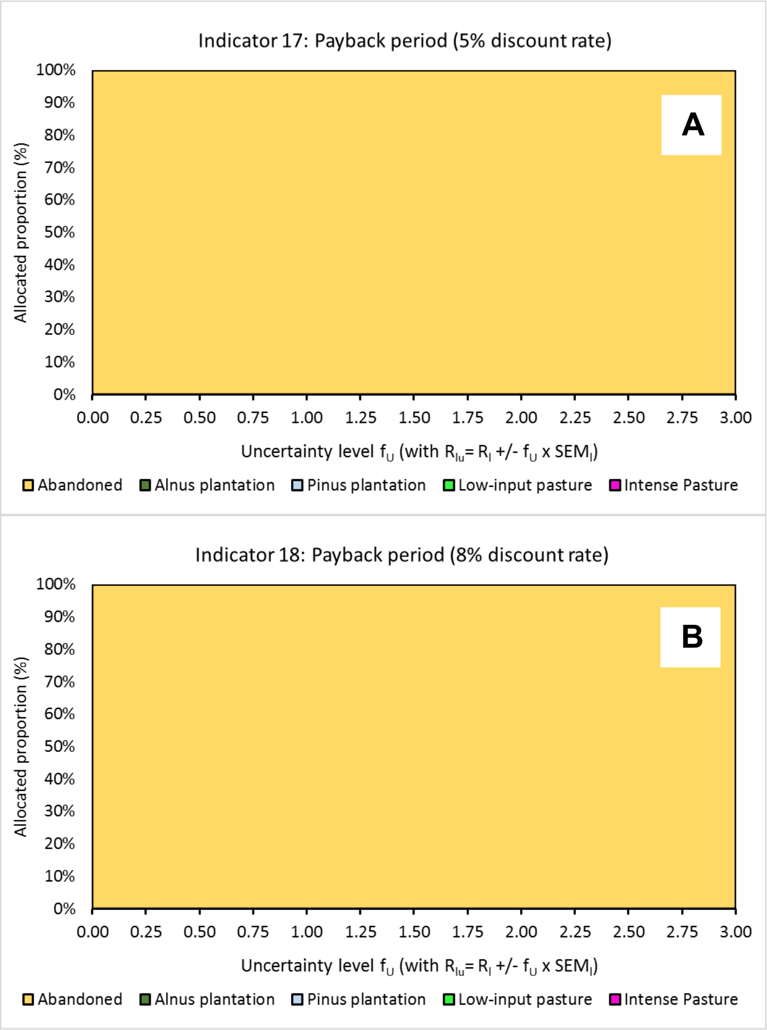

Supplementary Figure 7. Indicators for **Payback periods**[1] (time until the invested money is recovered) used for single objective optimization. The maximum distance to the 100% achievement level was minimized for 32 uncertainty scenarios considered at each uncertainty level 0.00 x SEM_l, 0.25 x SEM_l, …, 2.75 x SEM_l, 3.00 x SEM_l, with SEM_l being the standard error of the estimate for a land-cover option, *l*. When only one colour appears (A, B), the uncertainty considered was too small to suggest diversification.

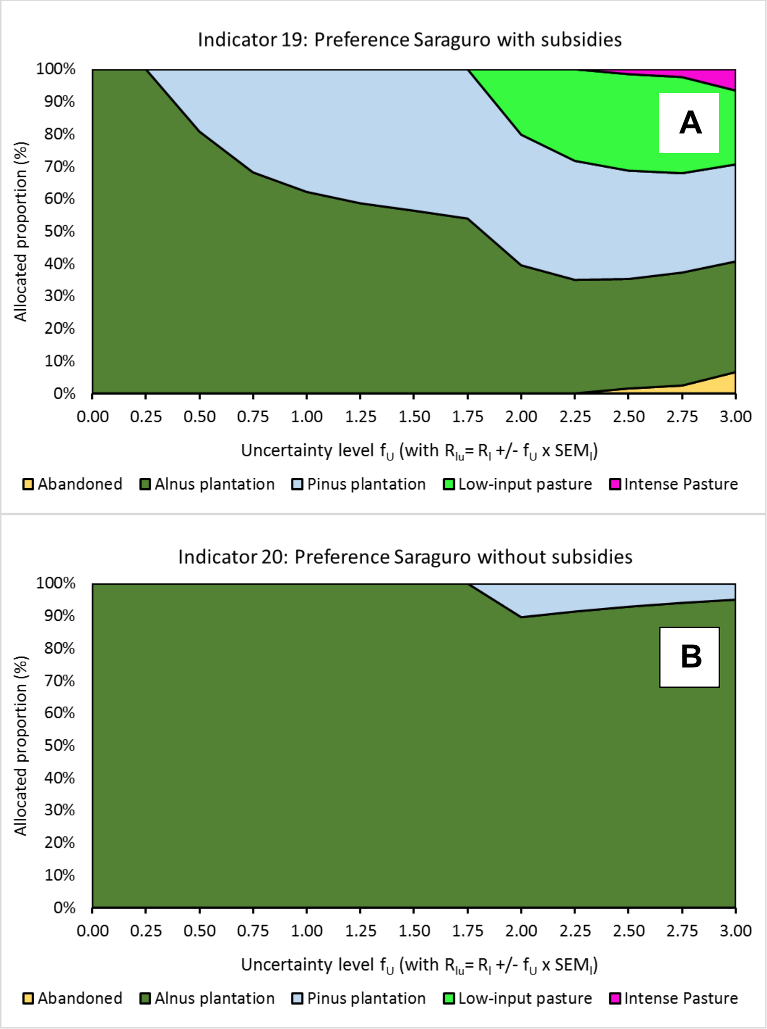

Supplementary Figure 8. Indicators for **Preferences of native Saraguros**[1] used for single objective optimization. The maximum distance to the 100% achievement level was minimized for 32 uncertainty scenarios considered at each uncertainty level 0.00 x SEM_l, 0.25 x SEM_l, …, 2.75 x SEM_l, 3.00 x SEM_l, with SEM_l being the standard error of the estimate for a land-cover option, *l*.

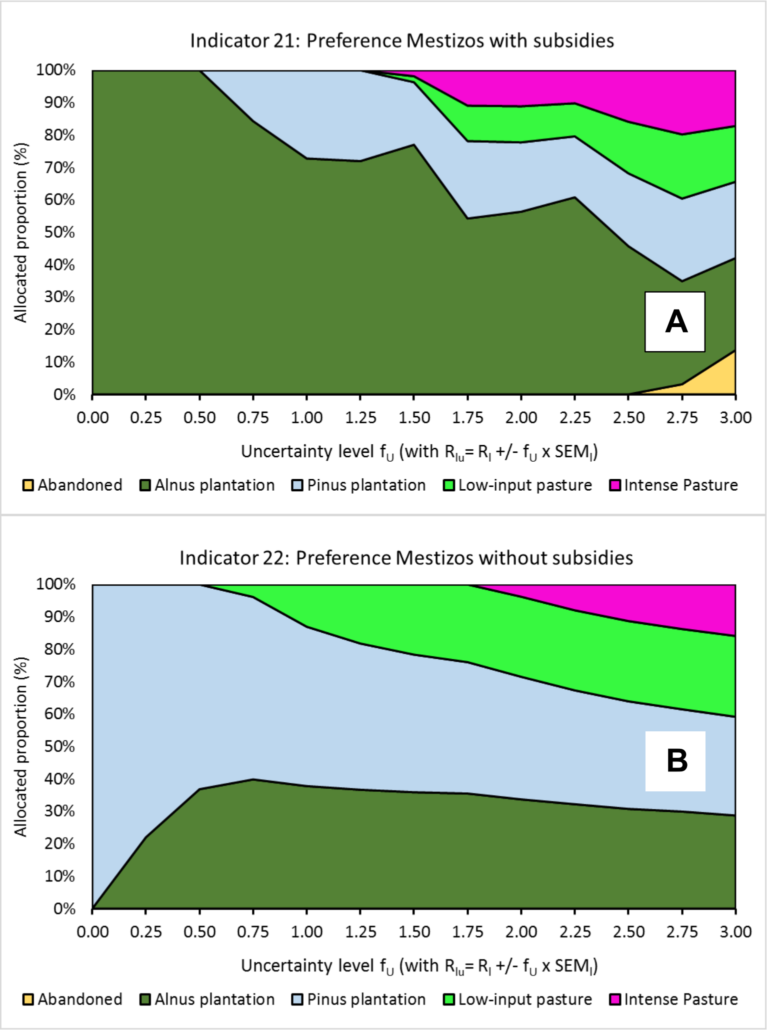

Supplementary Figure 9. Indicators for **Preferences of Mestizo Settlers**[1] used for single objective optimization. The maximum distance to the 100% achievement level was minimized for 32 uncertainty scenarios considered at each uncertainty level 0.00 x SEM_l, 0.25 x SEM_l, …, 2.75 x SEM_l, 3.00 x SEM_l, with SEM_l being the standard error of the estimate for a land-cover option, *l*.

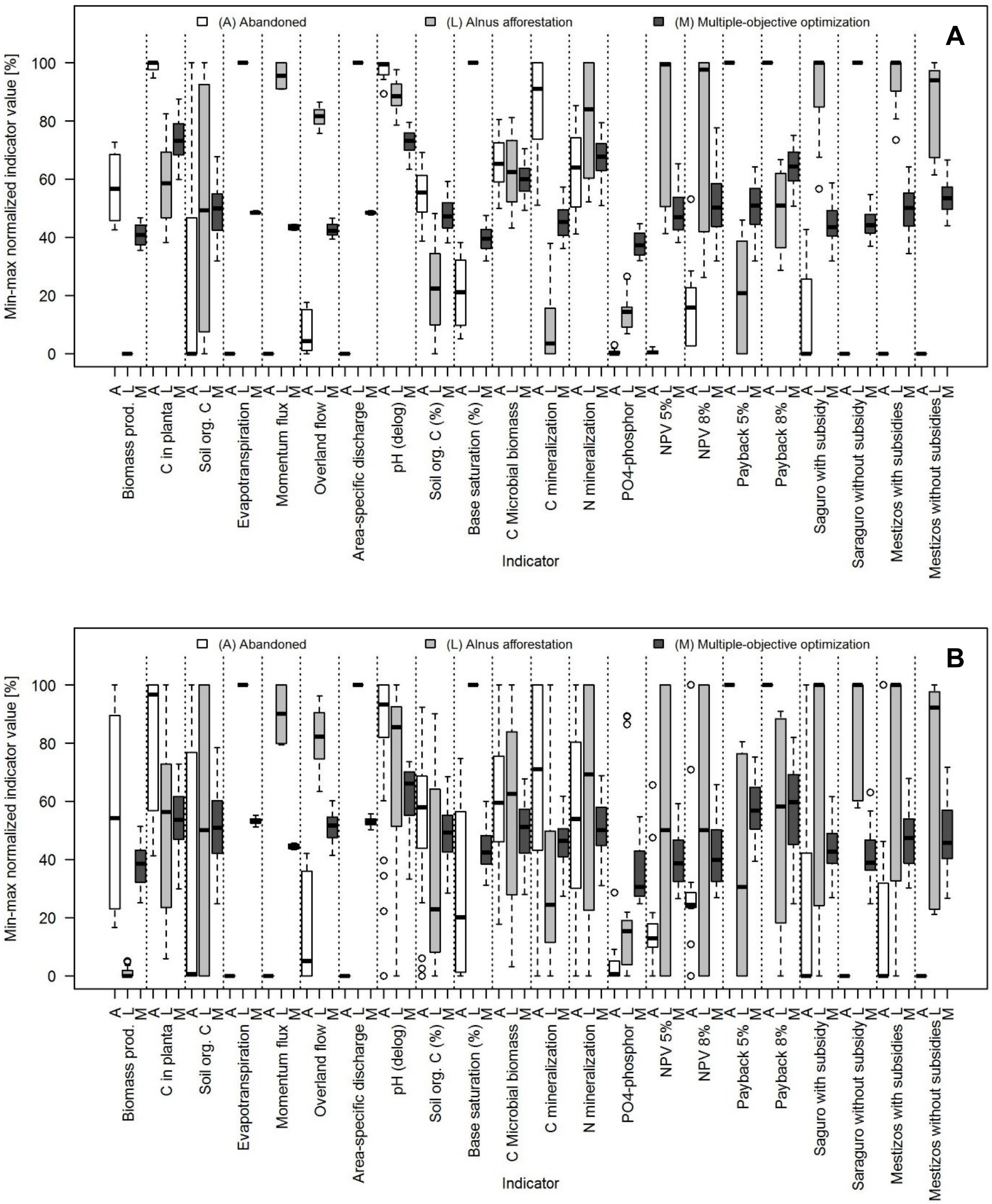

Supplementary Figure 10. Normalized landscape scale indicators (whiskers: minima and maxima, boxes formed by quartiles comprising the median) from multiple-objective optimization, compared with single land-cover options for altered uncertainty level of f_u=1 (A) and f_u=3 (B).

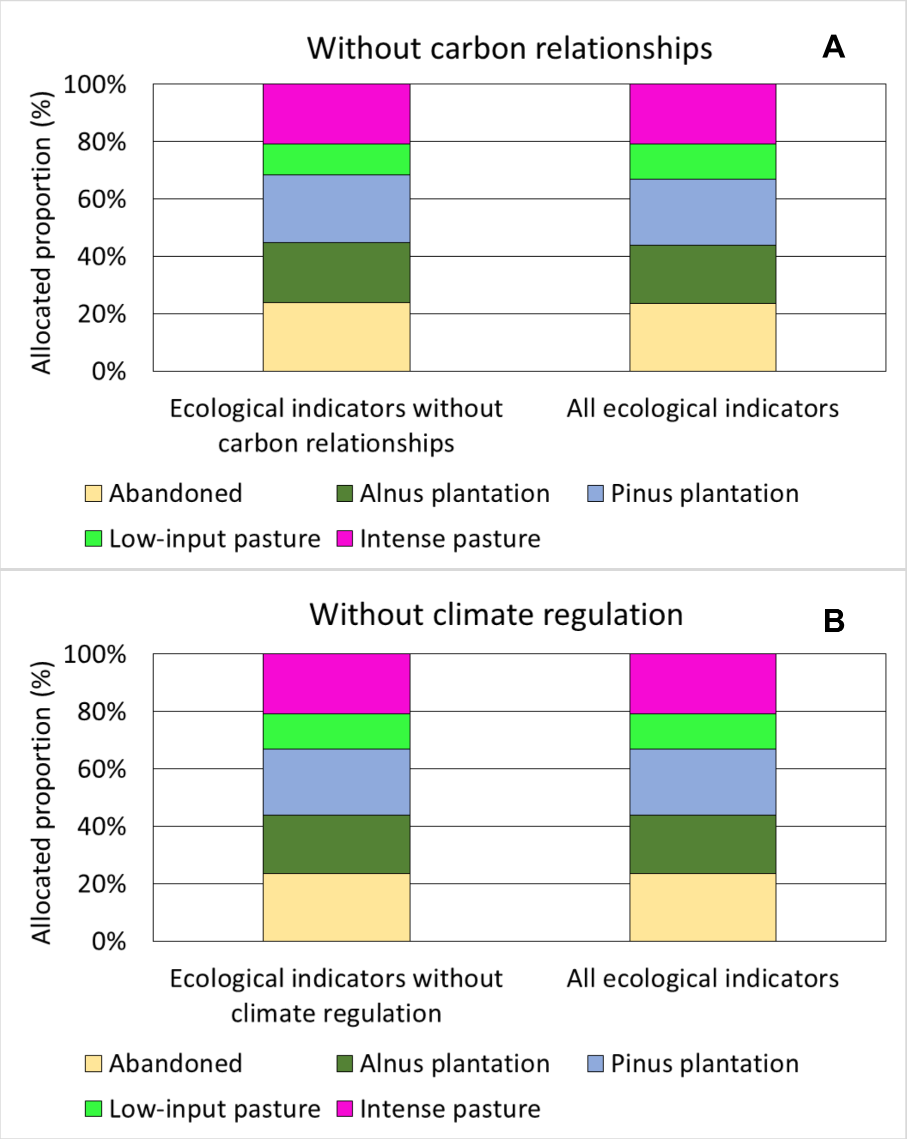

Supplementary Figure 11. Allocated percent proportion of land-cover options obtained from using various subsets of **ecological** indicators for optimization, each compared with the percent proportion obtained when using the full set of ecological indicators. Uncertainty spaces of the size “recorded coefficients ±2 x SEM_l” were used to create (A) and (B).

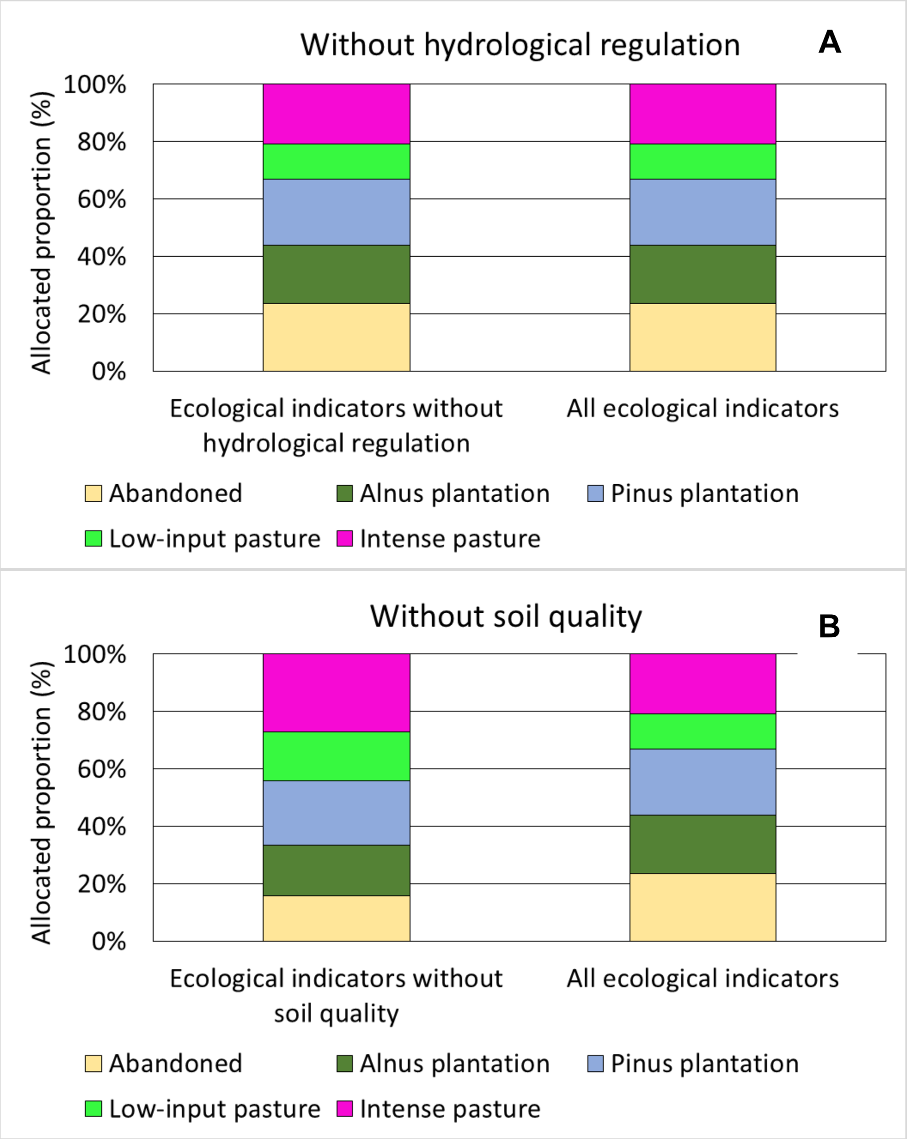

Supplementary Figure 12. Allocated percent proportion of land-cover options obtained from using various subsets of **ecological** indicators for optimization, each compared with the percent proportion obtained when using the full set of ecological indicators. Uncertainty spaces of the size “recorded coefficients ±2 x SEM_I” have been used to create (A) and (B).

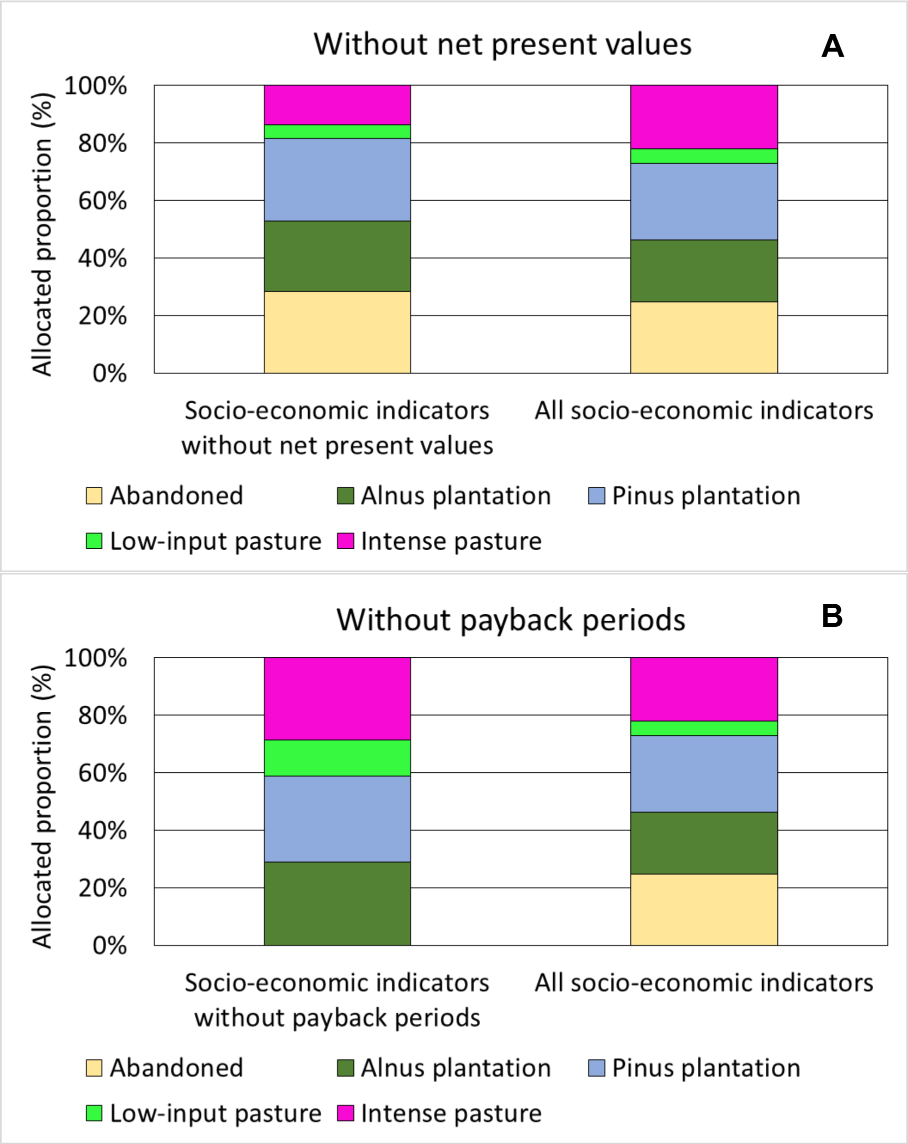

Supplementary Figure 13. Allocated percent proportion of land-cover options obtained from using various subsets of **socio-economic** indicators for optimization, each compared with the percent proportion obtained when using the full set of socio-economic indicators. Uncertainty spaces of the size "recorded coefficients ±2 x SEM_l" have been used to create (A) and (B).

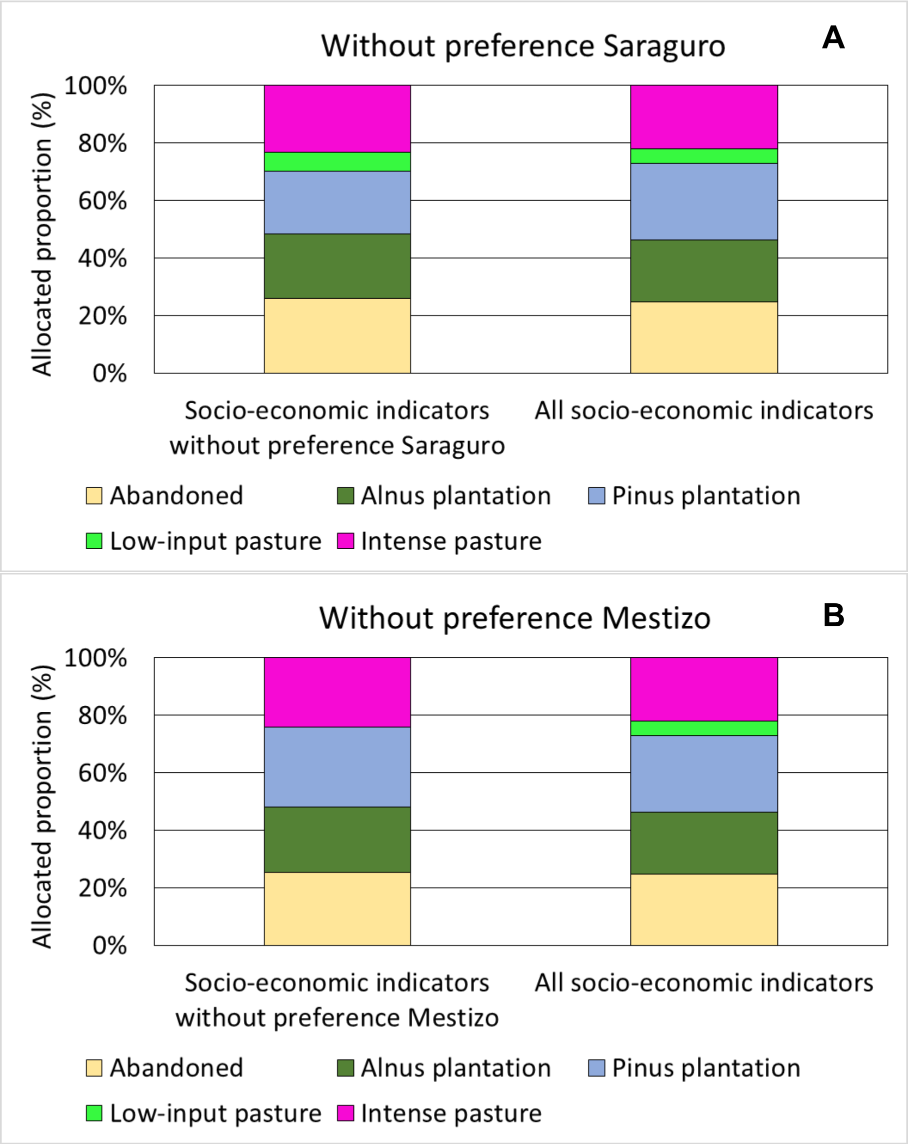

Supplementary Figure 14. Allocated percent proportion of land-cover options obtained from using various subsets of **socio-economic** indicators for optimization, each compared with the percent proportion obtained when using the full set of **socio-economic** indicators. Uncertainty spaces of the size “recorded coefficients ±2 x SEM_I” have been used to create (A) and (B).

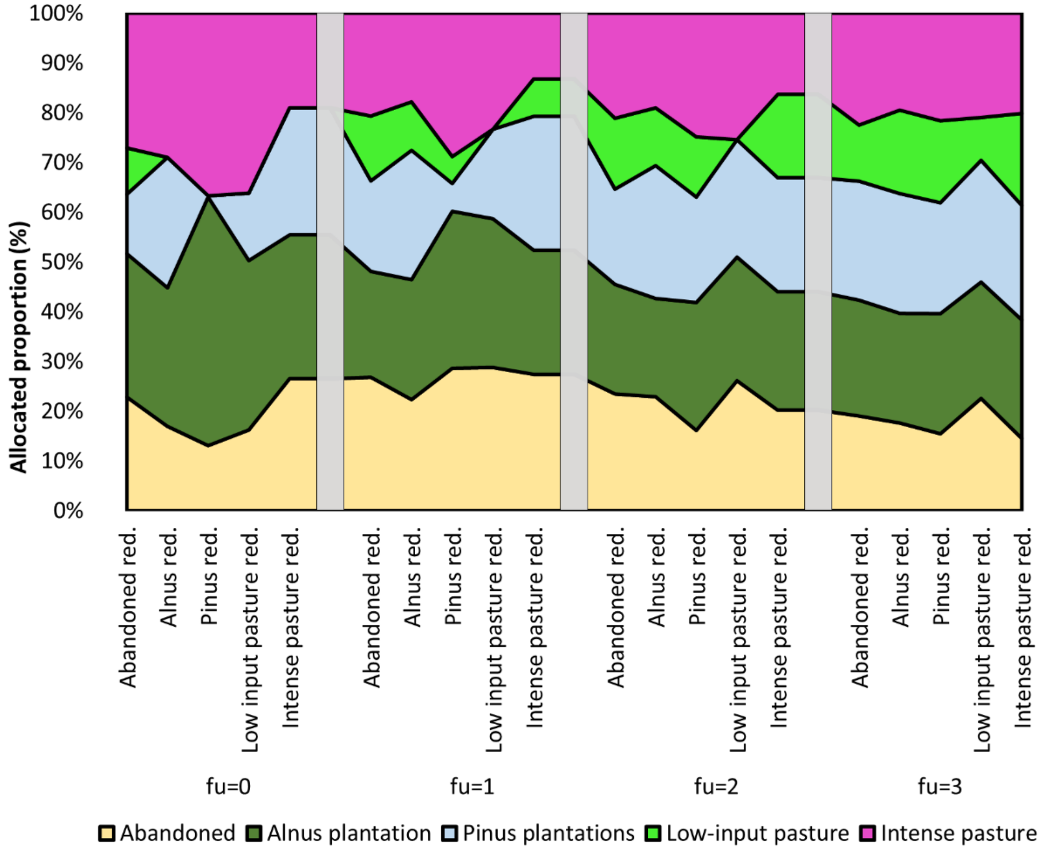

Supplementary Figure 15. Allocated proportions when expected performance of single land-cover options is reduced by 2 x SEM (standard error of the estimate), while performance is kept constant for the other land-cover options. Under f_u=0 only *Pinus* drops out, while for f_u=1 and 2 low-input pasture is excluded from the landscape portfolio.

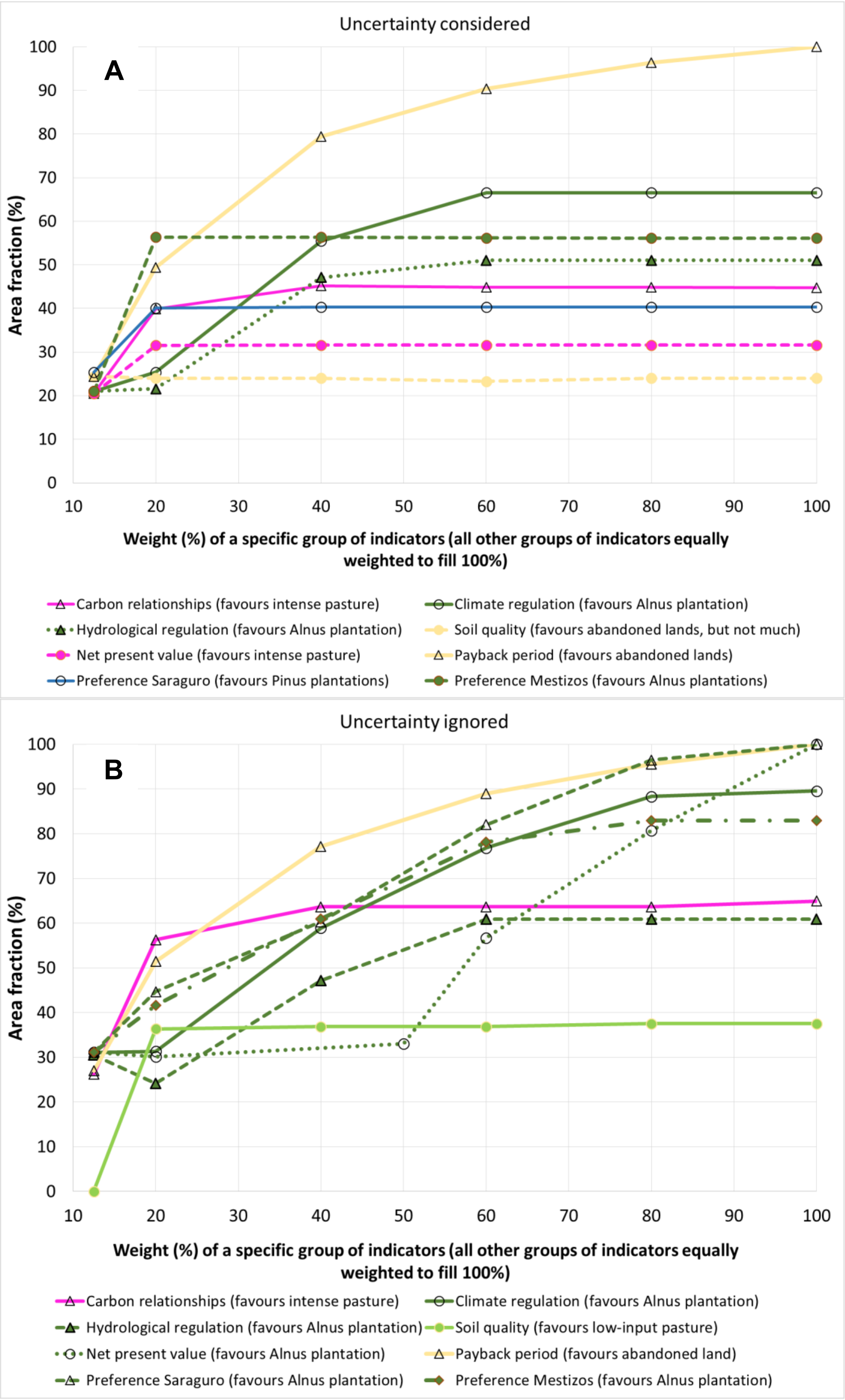

(see next page for figure caption)

Supplementary Figure 16. Allocated percent proportion to dominating land-cover types obtained from using increasing weights for specific groups of indicators. Uncertainty spaces of the size "recorded coefficients ±2 x SEM_I" were used to create (A), and uncertainty has been ignored for (B).

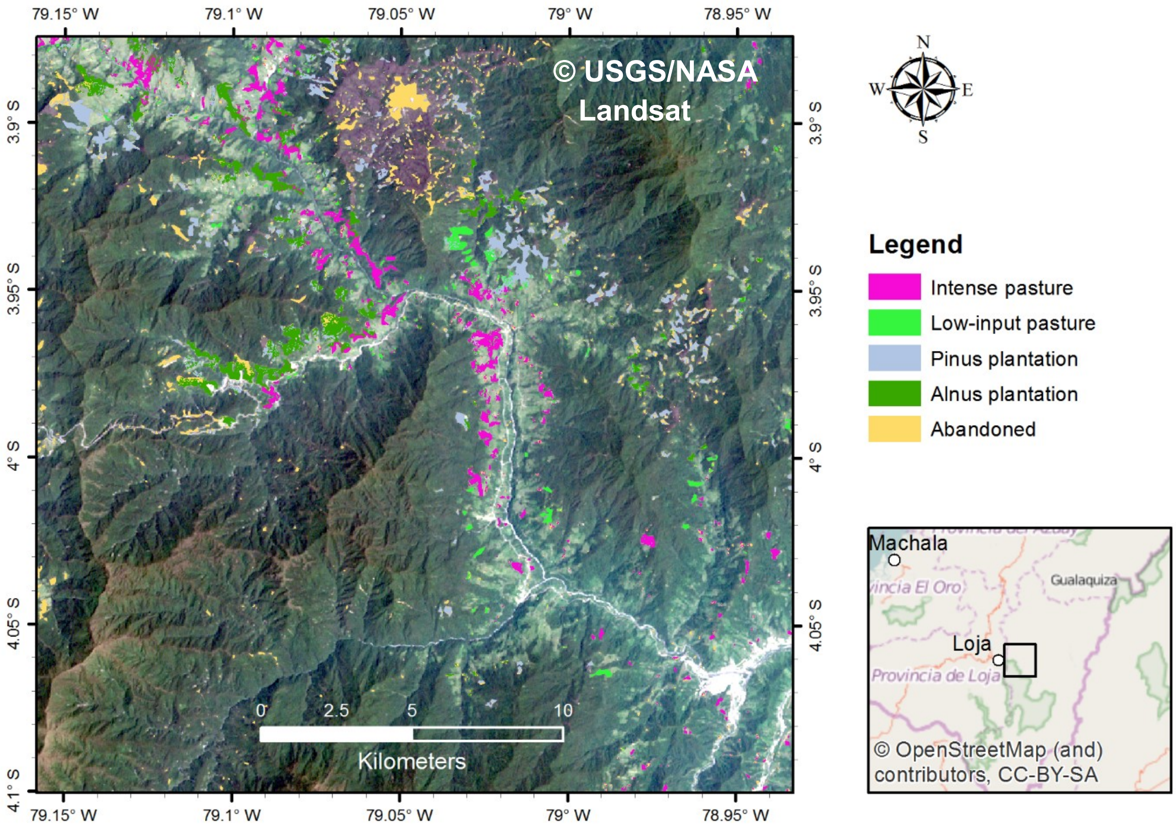

Supplementary Figure 17. One possible suggestion for forming priority zones: Distribution of a rehabilitation landscape portfolio (see Fig. 5, f_U=2, in main text) on currently abandoned areas in the San Francisco Valley, Ecuador, based on rule sets (see Supplementary Methods). Note that 24% of the initially abandoned areas remain abandoned to allow natural succession. The background image is a Landsat scene of 2001 in real composite colour (USGS/NASA Landsat); the purple shade surrounding the planned abandoned areas in the north originate from recent burnings.

Supplementary Tables

Supplementary Table 1. Spearman correlation between socio-economic indicators (grey shaded where exceeding 0.7)

		**Net present value**		**Payback period**		**Preference Saraguros**		**Preference Mestizos**	
		5%	8% interest	5%	8% interest	With	Without subsidies	With	Without subsidies
**Net present value**	5%	1	0.973	0.486	0.039	0.771	0.877	0.771	0.686
	8% interest		1	0.27	-0.189	0.701	0.822	0.703	0.501
**Payback period**	5%			1	0.878	0.551	0.534	0.823	0.964
	8% interest				1	0.125	0.143	0.487	0.724
**Preference Saraguro**	With					1	0.835	0.836	0.701
	Without subsidies						1	0.917	0.669
**Preference Mestizos**	With							1	0.908
	Without subsidies								1

Supplementary Table 2. Example for area-weighted absolute indicators derived with recorded/modelled (expected) values for optimal landscape portfolios (optimization based on all indictors)

Indicator	Unit	For uncertainty level f_U				Minimum	Maximum	Trend
		**0**	**1**	**2**	**3**			
Biomass production	Mg ha^{-1} per year	25.6	25.0	24.2	23.7	***7.7***	*50.0*	Decrease
Carbon in planta	Mg ha^{-1}	27.9	27.6	26.8	26.0	***12.5***	*33.0*	Decrease
Soil organic carbon	Mg ha^{-1}	92.0	91.7	92.0	92.3	***87.3***	*96.3*	Constant
Evapotranspiration	mm per year	1274	1253	1260	1284	***928***	*1597*	Constant
Momentum flux	kg m^{-1} s^{-2}	0.15	0.14	0.14	0.15	***0.02***	*0.29*	Constant
Overland flow	mm per year	57	57	56	56	*29*	***77***	Constant
Area-specific discharge		595	615	609	586	*283*	***927***	Constant
pH		4.1	4.1	4.0	4.0	***3.6***	*4.5*	Decrease
pH (delog)	mol per liter	0.000086	0.000086	0.000100	0.000099	***0.000032***	*0.000251*	
Soil organic carbon (SOC)	%	9.2	9.1	9.0	9.1	***6.8***	*11.7*	Constant
Base saturation		16.5	15.9	14.8	15.5	***6.4***	*30.4*	Constant
Carbon microbial biomass	mg kg^{-1}	1069	1046	1007	1012	***576***	*1359*	Constant
Carbon mineralization	g CO_2-C per kg SOC	3.44	3.49	3.50	3.47	***3.10***	*3.90*	Constant
N mineralization	mg N kg^{-1} per day	2.54	2.42	2.29	2.27	***1.00***	*3.00*	Decrease
PO_4-Phosphor	mg kg^{-1}	3.07	2.83	3.09	3.11	***0.50***	*6.00*	Constant
NPV 5%	US$ ha^{-1}	935	843	848	889	***0***	*1435*	Constant
NPV 8%		409	357	347	359	***-156***	*619*	Decrease
Payback 5%	Years	10	10	11	12	*0*	***18***	Increase
Payback 8%		11	11	13	14	*0*	***32***	Increase
Preference Saraguros with subsidy	Answers with preference rank 1 or 2	8	8	8	8	***4***	*14*	Constant
Saraguros without subsidy		9	8	8	9	***0***	*19*	Constant
Mestizos with subsidy		13	12	12	13	***5***	*19*	Constant
Mestizos without subsidy		10	10	11	12	***0***	*17*	Increase

Supplementary Table 3. Food and timber production of the landscapes considered (SEM: standard error of the mean)

	**Mixed landscape portfolios (24% abandoned, 21% *Alnus*, 25% *Pinus*, 10% low-input pasture, 20% intense pasture)**								**Deforestation based land use (100% low-input pasture after clearing of the natural forest)**			
**Product**	**Timber** [m^3 ha^{-1} yr^{-1}]				**Food** [milk in l; meat in kg ha^{-1} yr^{-1}]				**Food** [milk in l; meat in kg ha^{-1} yr^{-1}]			
Uncertainty level considered	Alnus	±SEM	Pinus	±SEM	Milk	±SEM	Meat	±SEM	Milk	±SEM	Meat	±SEM
0.0 x SEM_i	4.1	0.3	1.9	0.1	129.2	5.9	30.8	1.4	171	28.4	41	6.8
0.5 x SEM_i	4.3	0.3	1.8	0.1	106.8	4.9	25.4	1.2	171	28.4	41	6.8
1.0 x SEM_i	4.1	0.3	2.2	0.1	91.4	4.2	21.8	1.0	171	28.4	41	6.8
1.5 x SEM_i	3.6	0.3	2.5	0.1	129.8	7.2	30.9	1.7	171	28.4	41	6.8
2.0 x SEM_i	2.8	0.2	2.8	0.1	114.1	7.0	27.2	1.7	171	28.4	41	6.8
2.5 x SEM_i	3.1	0.2	2.6	0.1	120.6	7.5	28.7	1.8	171	28.4	41	6.8
3.0 x SEM_i	3.2	0.3	2.7	0.1	120.4	7.9	28.7	1.9	171	28.4	41	6.8
**Average**	**3.6**		**2.4**		**116.1**		**27.6**		**171**		**41**	

Supplementary Table 4. Opportunity costs of establishing rehabilitation options (differences of annualized net present values – the sum of all appropriately discounted net revenue flows over a 20-year period – between land use based on natural forest clearing and subsequent pasturing and rehabilitation options)

	**Opportunity costs (difference=deforestation based land use minus rehabilitation land cover) in US$ ha^{-1} yr^{-1}**					
Rehabilita-tion option	**NPV 5% discount rate**			**NPV 8% discount rate**		
	Difference	±SEM	Upper limit (+ 2 x SEM)	Difference	±SEM	Upper limit (+ 2 x SEM)
Abandoned	140	±27	194	151	±33	217
Alnus	25	±56	138	91	±52	195
Pinus	32	±55	141	93	±51	195
Low-input pasture	130	±29	189	168	±36	239
Intense pasture	55	±34	123	103	±40	182

Supplementary Methods

Indicators. We adopted the following description from Knoke *et al.* [1], who describe the procedures for recording or modelling the indicators in detail. We use 22 published indicators to thoroughly quantify the potential ecosystem functions and benefits provided by the land-cover types investigated. The indicators cover all categories of ecosystem services as defined by the Millennium Ecosystem Assessment[2]. They include supporting (biomass production, soil quality) and regulating functions (carbon, climate, and hydrology), as well as provisioning (timber, food) and social benefits (acceptance by the local people).

The indicator group *carbon relationships* characterises the uptake and accumulation of carbon - a primary ecosystem function that is a pivotal part of provisioning (for example, fodder for cattle or timber), regulating (storage of atmospheric carbon) and life supporting (organic matter to improve soil quality) ecosystem services. We use three indicators for the assessment of carbon relationships: “biomass production,” “whole plant-cover carbon accumulation” and “soil organic carbon”. *Climate regulation* is another important function of ecosystems, and the type and structure of the ecosystem directly influences the nature of surface-atmosphere exchanges. Large-scale land-cover changes alter both the microclimate and the climate regulation function of an ecosystem. The main drivers of this are changes in energy balance, surface roughness and evapotranspiration, all of which link atmospheric to hydrological functions. Here, we use “evapotranspiration” and “momentum flux” (turbulence production, an important land-atmosphere feedback parameter) to derive indicators for the intensity of surface-atmosphere exchanges. Because natural forest ecosystems usually show high exchange intensity, we consider a higher exchange intensity to be better than a lower intensity. *Hydrological regulation* of

the various land-cover types are crucial elements in assessing their potential for mitigating adverse effects of water (such as erosion), but also in controlling the quantitative supply of water. To quantify these effects we use the indicators “overland flow” and “area-specific discharge”. *Soil quality* is essential to maintain the long-term productivity, and thus the sustainability, of the provisioning services of our land-cover types. The chosen indicators are “pH value,” “soil organic carbon in percent,” “base saturation,” and “carbon in microbial biomass,” “carbon mineralization,” “nitrogen mineralization” and “PO_4-Phosphor”. These indicators vary in response to different land-cover types; they support plant productivity and contribute to soil biodiversity. *Economic indicators* of the rehabilitation options are imperative for analysing the likelihood that farmers will actually implement them. Thus, we use the simulated market value to quantify benefits from timber or food production. We use the “net present value” (NPV) and the “payback periods,” using two levels of discount rates (5%, and 8%) for each to quantify the economic benefits of each rehabilitation option. NPV is the sum of all appropriately discounted net revenues over a period of 20 years. Payback periods report the time necessary to recover the initial investment. *Social preference* serves as an indicator of the cultural benefit, for example the compatibility with traditional livelihoods, as well as their contribution to landscape aesthetics or preserving cultural heritage. Although people often consider both provisioning and regulating functions when expressing their preferences, they also tend to include intangible values of land use, which are largely determined by tradition, experience and personal preference. Because intangible cultural values are impossible to measure in ecological units, we use social acceptance as a meaningful proxy for cultural ecosystem benefits, benefits which existing approaches to assessing ecosystem services often ignore. We use

the “preference” of the land-cover types, with and without subsidies, from an evaluation expressed by indigenous Saraguro and Mestizo farmers.

Optimization. Here we document an alternative approach to multiple objective optimization (Supplementary equation (1)). The alternative formulation minimizes the largest distance between the maximum and the achieved level of ecosystem indicators directly through an appropriate objective function. However, this objective function is not smooth and, thus, we cannot solve it exactly[3]. Consequently, we based our allocation problem on constraints imposed on each of the 704 considered achievement functions, as described in the main text, to achieve an exact solution. The alternative formulation is as follows (here without any specific weighting of indicators and their difference to the maximum achievement level):

$$\min \max \left(D_{iu}\right)$$

subject to

$$R_{iu} = \sum_{l \in L} R_{liu} \cdot a_l$$
$$R_{liu} = R_{li} \pm f_U \cdot SEM_{il}$$
$$\sum_{l \in L} a_l = 1$$
$$a_l \geq 0$$

$$\text{if "more is better" for an indicator:}$$

$$D_{iu} = \frac{\max\left(R_{liu}\right) - R_{iu}}{\delta_{\max,\min}} \cdot 100 \qquad \forall i \in I \qquad \forall R_{liu} \in U_i$$

$$\text{if "less is better" for an indicator:}$$

$$D_{iu} = \frac{R_{iu} - \min\left(R_{liu}\right)}{\delta_{\max,\min}} \cdot 100 \qquad \forall i \in I \qquad \forall R_{liu} \in U_i \tag{1}$$

with

D_{iu}	Distance to the 100% achievement level for each normalized landscape level indicator, *i*, with 22 indictors and 32 uncertainty scenarios, *u*, being considered, representing all possible combinations of optimistic and pessimistic coefficients for each indicator
R_{iu}	Indicator (*i*) value at the landscape level for a specific uncertainty scenario (*u*)
a_l	Area proportion allocated to the land-cover option (*l*, representing 5 land-cover options), with *L* being the set of rehabilitation options considered
R_{liu}	Recorded indicator (*i*) value (R_{li}) for rehabilitation option (*l*) ± the considered deviation for a given uncertainty scenario (*u*) (see Figure 2)
U_i	Set of all uncertain indicator values; various U_i were considered, depending on the level of uncertainty considered (f_U) and the SEM of each indicator
i, I	Indicator (*i*) as a member of the set of indicators considered (*I*)
R_{li}	Originally recorded indicator value for a specific land-cover option (*l*) and indicator (*i*)
f_U	Factor to determine the level of the uncertainty deviation. f_U =0.000, 0.125, 0.250, …, 2.750, 2.875, 3.000
SEM_{il}	Standard error of the mean of the recorded indicator (*i*) and the land-cover option (*l*)
$\delta_{\text{max.min}}$	Range of indicator values within scenario *u*, $max(R_{liu})$-$min(R_{liu})$
$min(R_{liu})$	Minimum indicator value for each uncertainty scenario (*u*) among land-cover options (*l*)
$max(R_{liu})$	Maximum indicator value for each uncertainty scenario (*u*) among land-cover options (*l*)

Bringing rehabilitation plans into real landscapes – an example. To make the results of this study operational, and to minimize the risk of their implementation failing, the distribution of the landscape portfolios must be adapted to topographic, biogeochemical and sociological conditions. This is a complex subject which needs further research. Here, a dataset representing abandoned land areas in the San Francisco Valley[4], based on a Landsat scene from 2001 (total abandoned area: 3,601 ha; elevation: 920-2,714 m asl), was used to establish an exemplary rehabilitation plan (Supplementary Fig. 17). After preselecting areas <1 ha (which were then allocated to remaining as abandoned land; 11.9% of total area), we further subdivided the remaining areas (>1 ha) into five slope classes (0-12%, 13-25%, 26-40%, 41-70% and >70%) using the ASTER digital elevation model. The prioritization was then refined accounting for the elevation (five equal classes

within the complete range; the lower the better), the distance to roads (the smaller the better) and the area size (the larger the better) with descending priority. Subsequently we assigned the land-cover types (starting with the best sites for: intense pasture, low-input pasture, *Alnus, Pinus* and abandoned) until the allocated area proportions have been completed for each option. To make the implementation of the plan more feasible we revised the size of each resulting sub-polygon and allocated the areas < 1ha to a neighbouring rehabilitation option (same order as indicated above) within the same abandoned area, until a minimum size of 1 ha was achieved. Consequently, a slight shift of the final shares was obtained compared to the optimized shares (abandoned 24.5 vs. 24.2%; Alnus 21.4 vs. 20.6%; Pinus 25.6 vs. 24.9%; low-input pasture 8.4 vs. 10.4%; intense pasture 20.2 vs. 19.8%).

These landscape priority zones can be used to allocate financial and other incentives more effectively. The zoning may be combined with the REDD+ mechanism[5] or national programs such as the Ecuadorian “Socio Bosque”[6] for providing financial transfers to reward farmers for preserving their natural forests. Combining these transfers with rehabilitation will help increase the efficiency of forest preservation. For example, providing farmers living close to a National Park (in our case study those living near the Podocarpus National Park), with a gainful land-use activity[7] for their own degraded land could counter illegal logging inside the park. Consequently, existing programs should make transfers conditional to the implementation of specific rehabilitation activities on abandoned land. Supplementary Tables 3 and 4 list the possible opportunity costs for compensating farmers who rehabilitate their abandoned lands instead of clearing more forests. Offering contracts for property right contracts (possibly coupled with additional financial compensation) could be an effective alternative incentive for rehabilitation, an

option which appears particularly advisable in areas with chaotic property rights[1,8]. Finally, local experience should guide implementation of rehabilitation plans. In our case study such experience suggests, for example, that management practices should exclude fire to avoid favouring undesirable fire-resistant vegetation, such as bracken fern. Another important aspect to consider when planning rehabilitation in our study area is the dynamics of landslides, which occur in the abandoned areas mainly where roads have been constructed[9].

Achieved provisioning services and possible costs of rehabilitation (data from Knoke *et al.* [1]). The diversified landscape portfolio produces broadleaf and coniferous timber as well as milk and meat (Supplementary Table 3), while the rehabilitation landscape with *Alnus* would produce timber only at a level of 13.4 (±1) m^3 ha^{-1} yr^{-1}. If rehabilitation areas are regarded as potential replacements for deforestation based land use, their food production must be compared to the common food production system, which is deforestation based (business as usual, BAU). The BAU type of land use starts with the clearing of natural forest followed by pasture farming for milk and meat. The BAU scenario produces only food, but this at a level of 171 (±28.4) l ha^{-1} yr^{-1} for milk and 41 (±6.8) kg ha^{-1} yr^{-1} for meat. The amount of food attainable from the suggested rehabilitation areas (Supplementary Table 3) is around one third lower than the amount expected from the common deforestation-based land use. However, with intense pasture being an important component of rehabilitation of abandoned lands, the food production in the restored landscapes is still quite high. The productivity or the landscape portfolios reported in this study can thus be achieved with agricultural shares of only 19 to 32% of the total rehabilitated land. However, it must be kept in mind that about 1.33 hectares of

rehabilitated land would be needed to replace 1 hectare of deforestation based pastures to achieve the same level of food production.

If rehabilitation is to mitigate the pressure on the existing natural forests, the financial perspective of the farmers must also be considered. This requires calculating the opportunity costs of farmers who restore their abandoned lands instead of clearing natural forest for new pastures. Opportunity costs can be obtained by computing the annualized net present value of all future net revenues (annual return) for the various rehabilitation scenarios and by comparing this indicator with that of deforestation based land use (Supplementary Table 4).

The mean differences in annual return between the BAU land use and the single rehabilitation options ranges from US$ 25 to 168 ha^{-1} yr^{-1}. For a landscape portfolio to be rehabilitated according to the multiple-objective approach, average opportunity costs between ~US$ 70 and ~110 ha^{-1} yr^{-1} can be expected. The upper limits are two times the SEM of the differences. However, on a landscape level the diversified rehabilitation shows a much lower SEM compared with the BAU scenario, which should be considered as an advantage by risk-averse farmers. Considering the average opportunity costs instead of the upper possible limits therefore appears to be appropriate.

Supplementary references

1. Knoke, T. *et al.* Afforestation or intense pasturing improve the ecological and economic value of abandoned tropical farmlands. *Nature Commun.* **5,** 5612 (2014).
2. MEA. *Millennium Ecosystem Assessment - Ecosystems and human well-being: Synthesis* (Island Press, 2005).

3. Tamiz, M. Jones, D. & Romero, C. Goal programming for decision making: An overview of the current state-of-the-art. *Europ. J. Oper. Res.* **111,** 569–581 (1998).
4. Curatola Fernández, G. F. Obermeier, W. A. Gerique, A., López Sandoval, M. F. Lehnert, L. W. Thies, B. & Bendix, J. Land cover change in the Andes of Southern Ecuador: patterns and drivers. *Remote Sensing* **7(3)**, 2509-2542 (2015).
5. Pirard, R. & Belna, K. Agriculture and Deforestation: Is REDD+ Rooted In Evidence? *For. Policy Econ.* **21,** 62–70 (2012).
6. Mohebalian, P. M. & Aguilar, F. X. Additionality and design of forest conservation programs: Insights from Ecuador's Socio Bosque Program. *For. Policy Econ.* doi:10.1016/j.forpol.2015.08.002 (2015).
7. Knoke, T. Stimm, B. & Weber, M. Tropical farmers need productive alternatives. *Nature* **452,** 934 (2008).
8. Pohle, P. Gerique, A. Park, M. & Sandoval, M. in *Tropical Rainforests and Agroforests under Global Change* (eds Tscharntke, T. *et al.*) 477-509 (Springer, 2010).
9. Muenchow, J. Brenning, A. & Richter, M. Geomorphic process rates of landslides along a humidity gradient in the tropical Andes. *Geomorphology* **139–140,** 271–284 (2012).
